# Discrepancies between human DNA, mRNA and protein reference sequences and their relation to single nucleotide variants in the human population

**DOI:** 10.1093/database/baw124

**Published:** 2016-09-01

**Authors:** Matsuyuki Shirota, Kengo Kinoshita

**Affiliations:** 1 Graduate School of Medicine, Tohoku University, Sendai, Miyagi 9808575, Japan; 2 Tohoku Medical Megabank Organization, Tohoku University, Sendai, Miyagi 9808575, Japan; 3 Graduate School of Information Sciences, Tohoku University, Sendai, Miyagi 9808579, Japan; 4 Institute for Development, Aging and Cancer, Tohoku University, Sendai, Miyagi 9808575, Japan

## Abstract

The protein coding sequences of the human reference genome GRCh38, RefSeq mRNA and UniProt protein databases are sometimes inconsistent with each other, due to polymorphisms in the human population, but the overall landscape of the discordant sequences has not been clarified. In this study, we comprehensively listed the discordant bases and regions between the GRCh38, RefSeq and UniProt reference sequences, based on the genomic coordinates of GRCh38. We observed that the RefSeq sequences are more likely to represent the major alleles than GRCh38 and UniProt, by assigning the alternative allele frequencies of the discordant bases. Since some reference sequences have minor alleles, functional and structural annotations may be performed based on rare alleles in the human population, thereby biasing these analyses. Some of the differences between the RefSeq and GRCh38 account for biological differences due to known RNA-editing sites. The definitions of the coding regions are frequently complicated by possible micro-exons within introns and by SNVs with large alternative allele frequencies near exon–intron boundaries. The mRNA or protein regions missing from GRCh38 were mainly due to small deletions, and these sequences need to be identified. Taken together, our results clarify overall consistency and remaining inconsistency between the reference sequences.

## Introduction

Accurate sequences of the human genome and genes are fundamental resources for functional genomics and translational medicine. The human genome sequence was determined more than a decade ago (1), and has undergone several updates for refinement (2, 3). The human reference genome sequence refers to the one submitted and maintained by the Genome Reference Consortium (GRC) (3). Although many other groups have published alternative human genome sequences of separate individuals (4, 5), including those from different ethnicities (6, 7) and from the haploid genome of a hydatidiform mole (8), the reference genome has distinguished accuracy and coverage for difficult regions, including repeats and segmental duplications (9). However, these alternative genomes have played important roles in refining the reference genome, by identifying its erroneous regions (5, 8). Refinement of the reference genome is a continuous process, requiring efforts from both the experimental and bioinformatics fields (10–16). The latest reference genome, GRCh38, was published in 2013 and includes the results of many high-throughput sequencing efforts (17).

In addition to the genome sequence, the comprehensive inventory of human genes is essential for biological studies. These gene sequences, more precisely mRNA and protein sequences, can be derived either from the transcribed and protein coding sequences of the reference genome or from experiments based on independent biological materials. The former, the databases of human genes derived from the reference genome, include ENSEMBL and VEGA, and their sequences are consistent with the reference genome, because they are copied or translated from parts of the genomic sequence. The latter include RefSeq (18) and UniProt (19). RefSeq, more specifically its known RefSeq component, represents experimentally validated mRNA transcript sequences, along with protein sequences translated from the coding regions of the mRNA sequences. UniProt contains only amino acid sequences of proteins, together with their functional annotations. These independent human gene sequence databases are quite valuable, because they provide quality control for the protein coding regions of the reference genome. It has been noted that some of the RefSeq and UniProt sequences are inconsistent with the sequences expected from the coding regions on the human genome (20, 21). The main reason for these differences is assumed to be due to polymorphisms or rare variations in the human genome, because different experiments to determine the reference sequence of the same gene may use different polymorphic alleles. However, other explanations exist for the cause of these differences. One possibility is the erroneousness or incompleteness of either the reference genome or gene sequences, which have sometimes been recognized for both the genome and gene sequences. It is also possible that some mRNA or protein sequences are inconsistent with the genome sequence within a single biological material due to post-transcriptional and post-translational modifications, such as RNA editing. Finally, incorrect annotations of gene loci, which sometimes occur around exon–intron boundaries, can lead to apparent differences between gene and genome sequences. Although the differences in the RefSeq mRNA and UniProt protein sequences from the reference genome have been pointed out several times, the cause of this discordance has not been well characterized.

The last decade has seen a remarkable improvement in sequencing technology, which facilitated the re-sequencing of a huge number of human individuals’ genomes (22, 23). Two major sources are the 1000 genomes (1K genomes) (24, 25) and the exome sequencing project of > 6500 individuals by the National Heart, Lung and Blood Institute (ESP6500) (26, 27). These large-scale analyses provide a rich resource of millions of genomic variations, together with their alternative allele frequencies (AAFs) in the coding regions. Thus, if the reference sequences for a single gene from two databases conflict with each other, it is now possible to determine whether they are known polymorphic sequences and, if so, which sequence is more suitable for the human reference by way of majority vote. Although the differences between genomic and mRNA reference sequences have sometimes been noted (20), the frequency information has not been taken into account. In addition, RNA sequencing has been performed for some of the individuals in the 1000 genomes (28), which enables the analysis of post-transcriptional modifications by comparison of the genomic and transcriptomic sequences in the same individuals. Furthermore, the comparison of protein coding sequences (CDS) on non-synonymous polymorphisms can be extended to include other protein databases, such as UniProt and PDB, which have not been considered despite their importance in functional analysis. Direct comparisons of the amino acid sequences derived from the three databases, the reference genome, RefSeq and UniProt, will help us to understand how much they are consistent and how much and why they differ with each other.

In this study, we employed GRCh38, RefSeq and UniProt as the sources of the DNA, mRNA and protein reference sequences, respectively. We compared the protein coding sequences of GRCh38 and RefSeq and the corresponding amino acid sequences of UniProt, and related the differences between them to the genomic coordinates to determine whether they can be described by human genomic variations with known allele frequencies. We discuss why these differences occur and how these differences affect biological researches.

## Material and methods

### Reference sequences of the three databases

We used the following reference sequences. (1) Human genome GRCh38 (2013) was downloaded from the University of California Santa Cruz (UCSC) website (29), and it contains 22 autosomal, two sex and one mitochondrial chromosomes, 261 alternative loci and 42 unlocalized and 127 unplaced scaffolds. (2) Human mRNA sequences were downloaded from NCBI RefSeq on September 30, 2014. We employed the known RefSeq records, which are supported by curation and primary transcript evidence, and they consist of 38 095 mRNA transcript entries, with IDs starting with ‘NM_’, for 19 238 genes (18). (3) Human reference protein sequences were downloaded from UniProt on December 22, 2014 (19). They consist of 41 982 manually curated SwissProt sequences and 46 735 automatically processed TrEMBL sequences. We used both SwissProt and TrEMBL entries, considering the splicing isoforms separately, totalling 88 717 reference sequences.

### Comparison of the RefSeq mRNA sequences and GRCh38 genome sequences

We first compared the human mRNA sequences in RefSeq with the genomic sequences within the corresponding loci of the human reference genome GRCh38. We restricted the comparison to the protein coding sequences (CDS), because the 3′ and 5′ untranslated regions are usually less conserved than the CDS. The genomic regions encoding proteins are unambiguous for the majorities of the transcripts, but some uncertainties still exist for the remaining transcripts, especially in the correct definition of exon–intron boundaries. To determine the reliable exonic regions of each mRNA, we employed the following three criteria: (i) the gene loci annotation provided by the UCSC database (downloaded as refFlat.txt) (29), (ii) the annotations provided by the consensus CDS (CCDS) database (21) and (iii) the alignment of the mRNA sequences to the genome by the BLAT program (30) with default options. Since UCSC and BLAT may annotate more than one locus for a single mRNA, we selected the best matching locus for each mRNA with the smallest number of mismatching and missing bases in the CDS regardless of whether the locus is on a canonical (i.e. 22 autosomal and two sex) chromosome or on an alternative scaffold. If the number of mismatching and missing bases in the CDS is the same between a locus in a canonical chromosome and a locus in an alternative scaffold, the one in the canonical chromosome was used.

This comparison classified the transcripts into four classes by each annotation, as follows:

class 1: the coding sequences of mRNA and the reference genome *exactly matched*,
class 2: *some substitutions* are observed but all of the bases in mRNA CDS are aligned to GRCh38
class 3: some *deletion* bases are observed in mRNA aligned with GRCh38,
class 4: the mRNA has no matching locus in GRCh38 (*missing mRNA*).

For those mRNAs that were assigned to different classes by different methods, we used the CCDS class if available, because it is the most intensively curated. If the CCDS annotation was not available (CCDS class 4), we selected the better class between the UCSC and BLAT annotations for each mRNA.

### Assignment of allele frequency of known single nucleotide variations

Variant Call Format (VCF) files of the 1000 genomes (phase 3) were downloaded from the 1000 genomes website (http://www.1000genomes.org/) (24). Those of the ESP6500 study were downloaded from the Exome Variant Server, NHLBI GO Exome Sequencing Project (ESP) Seattle, WA (URL: http://evs.gs.washington.edu/EVS/) [accessed November 2014] (26). As these VCF files were based on the GRCh37 coordinates, we obtained the positions of the variants on the GRCh38 coordinates using the liftOver program. The reference and alternative allele frequencies were taken from the VCF files.

It should be noted that some regions in GRCh37 are inverted in GRCh38. To check if a variant is located in an inverted region between GRCh37 and GRCh38, we compared the sequences of the 21 nucleotides (ten nucleotides for each side) around the variant position for the GRCh38 forward strand and the GRCh37 forward and reverse strands. If the 21 nucleotide sequence surrounding the variant in the GRCh38 forward strand is more similar to that in the GRCh37 reverse strand than to that in the GRCh37 forward strand, then the variant is judged to be located in the inverted region and the reference and alternative bases in the VCF file were changed to their complementary ones, respectively.

After taking the inversion of the variant position into account, we checked if the reference and alternative bases of the position are switched with each other from GRCh37 to GRCh38. If the switch occurred, then the allele frequencies of the reference and alternative alleles were changed with each other from GRCh37 to GRCh38.

### Alignment of RefSeq and UniProt protein sequences and identification of substituted amino acids

The protein coding sequences of the RefSeq mRNA entries, with IDs starting with ‘NM_’, were translated to yield protein sequences as described in the GenBank formatted file, and confirmed to be consistent with their corresponding RefSeq protein sequences, with IDs starting with ‘NP_’. Special care must be taken for the following irregular translation patterns described in the GenBank formatted file: (i) frame-shifting of translation can occur within CDS by ribosomal slip, (ii) codon UAG may not be recognized as a termination codon, but translated to selenocysteine and (iii) non-ATG first codon can be translated to methionine. We first compared these 38 095 RefSeq protein sequences with 88 717 UniProt sequences, and found that 34 600 matched perfectly to at least one of the UniProt protein sequences. We used the BLAST program to search the remaining 3495 RefSeq protein sequences in the UniProt sequences. Among them, 1786 had similar sequences in UniProt proteins at the threshold of 95% sequence identity and had the same length. We considered the substitutions between these 1786 RefSeq-UniProt sequence pairs of the same length to be the candidates for those non-synonymous variants for which RefSeq and UniProt represent different alleles, and examined whether the 1K genomes can explain these substitutions. Other amino acids in these 1786 RefSeq protein sequences and all of the amino acids in the 34 600 RefSeq proteins, which matched UniProt entries, were regarded as matched between RefSeq and UniProt. The amino acids of the remaining 1079 RefSeq proteins that could not be matched or aligned within our threshold to UniProt sequences were not considered for the analysis of the concordance between GRCh38, RefSeq and UniProt (Figure 3).

### Identification of variant positions from RNA-seq reads

The binary sequence alignment matching (BAM) files of the mRNA-sequencing for 462 individuals included in 1000 genomes were downloaded from the GEUVADIS website (http://www.geuvadis.org/web/geuvadis/) (28). We examined the RNA-seq reads covering the substitution positions between RefSeq mRNA and GRCh38, which could not be explained by known SNVs in the 1K genomes or ESP6500. The genotypes of these 462 individuals determined by DNA sequencing were considered to be homozygous to the reference allele for the substituted positions, because no variants were reported for them. For all of the reads that match each of the substituted positions without base insertions and with mapping quality ≥30, we counted the numbers of GRCh38 and RefSeq mRNA bases with a base quality threshold of 30. The bases that match RefSeq mRNA but not GRCh38 indicate that post-transcriptional modifications occur for those mRNA molecules.

### Assignment of PDB structures for non-synonymous SNVs

We searched 75 982 non-redundant (i.e. not identical with each other) sequences of the protein chains in the PDB against the RefSeq protein sequences with the BLAST program, at the thresholds of *E*-value < 0.01 and sequence identity > 95%. Significant RefSeq-PDB protein sequence pairs sharing the same gene name and the same taxonomy (*Homo sapiens*) were realigned with the needle program (31). For each of the non-synonymous SNVs of 1K genomes that could be liftOvered to the GRCh38 coordinates, the amino acid changes in the RefSeq protein sequences were first identified, and then we examined whether any protein chains in the PDB include the coordinates of the corresponding reference or alternative amino acids.

## Results and discussion

### Comparison of RefSeq mRNA sequence with the reference genome

We first classified 38 095 RefSeq human mRNAs into four classes according to the alignment of the CDS sequences, as described in the ‘Methods’ section. The mRNAs in class 1 matched completely with GRCh38 over the CDS, those in class 2 included some base substitutions from GRCh38, those in class 3 had at least one base missing from GRCh38, and those in class 4 did not have a gene locus assignment (see ‘Methods’ section for details). Finally, we determined all mRNA classes uniquely, and obtained 36 281, 1772, 41 and 1 mRNAs in classes 1, 2, 3 and 4, respectively. The number of mRNAs classified for each class by each method is summarized in Supplementary Table S1, and the classification inconsistencies between UCSC and CCDS are discussed in the Supplementary Discussion.

In the following parts of this manuscript, we first discuss the mismatching bases between RefSeq mRNA and the reference genome (class 2), and then summarize the bases in mRNAs deleted from GRCh38 (class 3).

### Differences in substitutions (class 2)

We first explored base mismatches between 1772 class 2 RefSeq mRNAs and the GRCh38 reference genome. By removing the redundancy occurring in the isoforms of the same gene, the mismatches were grouped into 1179 genomic substitutions according to the coordinates on GRCh38, with 640 synonymous and 539 non-synonymous. Among them, 87% (555) of the synonymous and 90% (490) of the non-synonymous substitutions are observed in either the 1K genomes or the ESP6500 studies with allele frequencies assigned. Three synonymous and four non-synonymous substitutions match triallelic sites reported in the 1K genomes, in which RefSeq represents one of the two alternative alleles. These results suggest that the majority of the differences between GRCh38 and RefSeq mRNA originate from natural variations in the human genome. Other differences may indicate that RefSeq mRNA represent rare variations, which have not been explored in the two population genomics studies, that it includes some erroneous bases in its sequences, or that the mRNA and genome alignment includes errors. We hereafter refer to such genomic variants that occur when the reference genome represents different alleles from the RefSeq mRNA as *discordant variants*.

**Figure 1.**
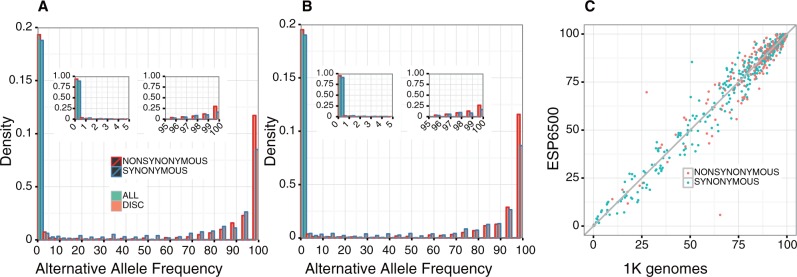
Alternative allele frequencies (AAFs) of the genomic positions in which RefSeq mRNA represents the alternative allele. (A and B) Density distributions of alternative allele frequencies for each 5% frequency interval based on (A) 1K genomes and (B) ESP6500 data. Green and orange bars represent the distributions for all of the SNVs in the data set and those of the SNVs corresponding to the difference between RefSeq mRNA and GRCh38, respectively. Red and blue borders show non-synonymous and synonymous substitutions, respectively. Insets represent the densities for each 1% frequency interval within 0–5% and 95–100%. (C) AAFs for the discrepancy loci between 1K genomes and ESP6500 data. Synonymous and non-synonymous substitutions are shown in green and orange, respectively.

We compared the distributions of alternative allele frequencies (AAFs) for the discordant variants and those for all of the variants in the CDS, based on the 1K genomes and ESP6500 biallelic sites. Both the 1K genomes (Figure 1A) and ESP6500 (Figure 1B) distributions show an abundance of low frequency (<5%) alternative alleles in all CDS variants (green bars), while the alternative alleles are the majorities in the discordant variants (red bars). A finer inspection of the variants with low (<5%) and high (>95%) AAFs revealed that the distributions of discordant and all variants peak at the highest (99–100%) and lowest (0–1%) AAF bins, respectively (Figure 1A and B, insets). The AAFs of discordant variants from the 1K genomes and ESP6500 correlate well (Figure 1C), supporting the generality of the result. This trend was the same for both synonymous and non-synonymous variants. These results suggest that the mismatches between GRCh38 and RefSeq mRNA are derived from the fact that the reference genome represents minor or sometimes rare alleles at a considerable number of genomic positions.

**Figure 2.**
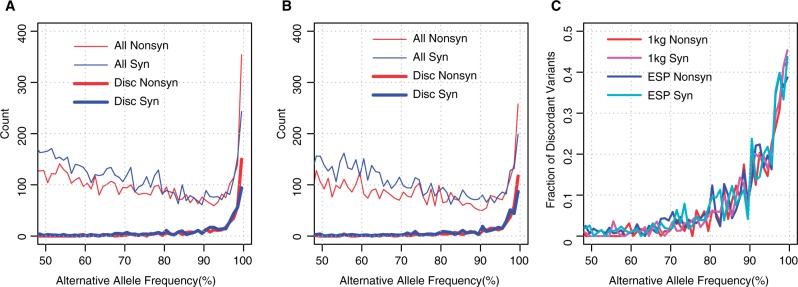
Counts of variants with large alternative allele frequencies. (A and B) The number of variants for each 1% of alternative allele frequency is plotted for (A) 1K genomes and (B) ESP6500. Red and blue lines indicate non-synonymous and synonymous variants. Thin and thick lines indicate all and discordant variants. (C) Fraction of discordant variants for each 1% alternative allele frequency bin. Red and magenta lines indicate non-synonymous and synonymous variants for 1K genomes, respectively, and blue and cyan lines are those for ESP6500, respectively.

We then examined the fraction of the entire high AAF variants that consists of the discordant variants. The counts of variants with >50% AAFs for each 1% bin based on the 1K genomes and ESP6500 data are shown in Figure 2A and B, respectively. The number of all variants (thin lines) declines with AAF until 90%, and then grows rapidly between 90 and 100%, while the number of discordant variants (thick lines) is almost 0 until 90% AAF, and increases sharply between 90 and 100%. The fraction of discordant variants increases to as much as 40% of all variants in AAF > 99% (Figure 2C). Thus, although most of the discordant variants have high AAFs, they are not the majority of the high AAF variants in the genome and there still are several hundreds of variants with high AAFs, for which both the GRCh38 and RefSeq mRNA databases represent the minor allele as the reference. Some of these may be ‘private’ minor alleles, which derive from the small number of individuals who provided their biological samples for the generation of the reference genome sequence. To clarify those discordant variants, we further checked the consistency between the RefSeq mRNA and UniProt databases.

### Non-synonymous discordant variants in UniProt sequences

For non-synonymous discordant variants between GRCh38 and RefSeq mRNA, it is possible to determine which of the two alleles is supported by the UniProt reference protein sequence. We determined the allele of the UniProt reference protein for 515 out of 539 non-synonymous discordant variants. Overall, the UniProt sequence supported GRCh38 and RefSeq mRNA at 410 and 105 substitutions, respectively. The majority of the substitutions were known variants in the 1K genomes: 400 out of 410 substitutions where UniProt supports GRCh38 and 72 out of 105 where UniProt supports RefSeq mRNA. As described in the previous section, the majority of the discordant variants between GRCh38 and RefSeq mRNA show high alternative allele frequencies (Figure 1), indicating that the RefSeq mRNA alleles are usually the major ones. This high rate of GRCh38 alleles represented by UniProt may indicate that some minor alleles are used as the UniProt reference sequence, presumably because they are represented by the reference genome. To examine this possibility and to understand the allele frequency distributions of the discordant variants more precisely, we classified the non-synonymous SNVs observed in the 1K genomes according to the concordance of the alleles between GRCh38, RefSeq mRNA, UniProt protein and GRCh37, the previous reference genome. GRCh37 was included because numerous minor alleles in it were changed to the major ones in GRCh38, and this previous reference genome may have some influence on the RefSeq mRNA and UniProt reference sequences.

### Alternative allele frequency distribution for each discordance type

**Figure 3.**
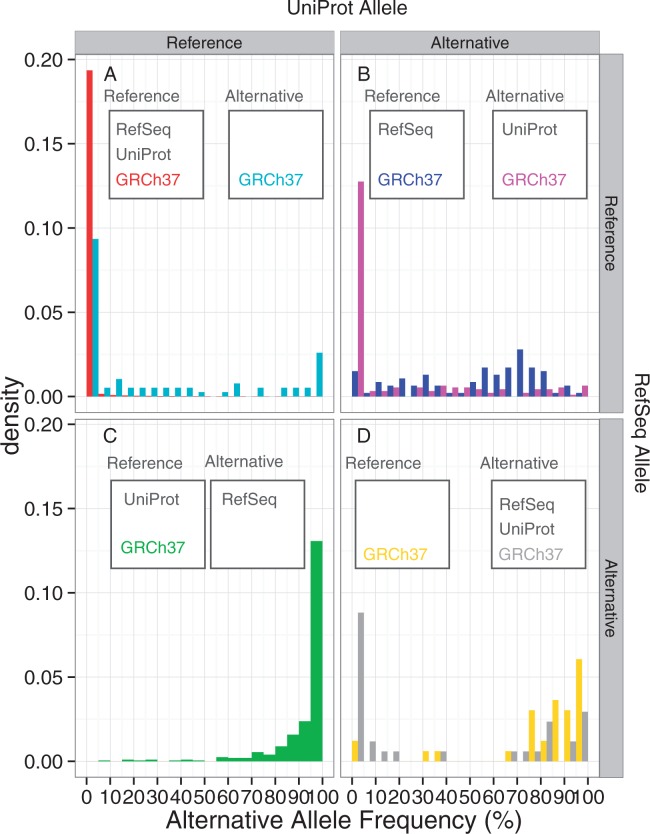
The alternative allele frequencies of the 1K genomes SNVs, classified by the concordance and discordance statuses between GRCh38, RefSeq mRNA, UniProt protein and GRCh37. The reference allele is defined by the GRCh38 sequence. (A) Both RefSeq and UniProt represent the reference allele, (B) RefSeq represents the reference allele but UniProt represents the alternative allele, (C) RefSeq represents the alternative allele but UniProt represents the reference allele, (D) both RefSeq and UniProt represent the alternative allele. The two histograms in panel A, B and D indicate that GRCh37 represents the reference (left) and alternative (right) alleles. On the other hand, panel C has only one histogram, because no SNVs were found for which RefSeq and GRCh37, but not UniProt, represent the alternative allele.

Table 1 shows the number of non-synonymous SNVs in the 1K genomes for which the reference alleles of RefSeq and UniProt could be determined. The majority (584 059, 99.9%) of the SNVs are those variants in which the reference alleles of GRCh38, RefSeq, UniProt and GRCh37 coincide with each other. Among the remaining SNVs, in 404, 93 and 76 variants, RefSeq, UniProt and GRCh37, respectively, represent the alternative allele as compared to GRCh38. UniProt and GRCh37 in 186 variants and RefSeq and UniProt in 33 represent the alternative alleles as the reference, whereas no position was found in which RefSeq and GRCh37, but not UniProt, represent the alternative allele as the reference. In 35 variants, RefSeq, UniProt and GRCh37 represent the alternative allele of GRCh38 as their references. We then examined the alternative allele frequencies of the positions in each category from the 1K genomes study (Figure 3A–D). In the large majority of the all-concordant cases (Figure 3A, red), almost all SNVs are low frequency variants, with an allele frequency < 5%. In those positions where only GRCh37 is the alternative allele from GRCh38, although the majority has low AAFs, some positions have high AAFs (Figure 3A cyan). This indicates that upon the update of the reference genome, many minor alleles are corrected to the major alleles, whereas some major reference alleles are changed to the minor ones in a few cases. In contrast, the alternative allele dominates in most of those variants where the RefSeq mRNA alone uses the alternative allele of GRCh38 as their reference (Figure 3B, green). This result is consistent with Figure 1, reflecting that the RefSeq mRNA reference alleles are more likely to be the major ones among the discordant variants. In those variants where UniProt protein is different from GRCh38 and RefSeq mRNA, the distribution of AAF changes by the GRCh37 allele type (Figure 3C). If GRCh37 is the same as GRCh38 (blue), then the allele distribution is relatively uniform, indicating that these variants are polymorphic ones. However, if the GRCh37 allele is the same as the UniProt protein one but different from that of GRCh38 (magenta), then alternative allele frequency is low, suggesting that the old reference genome and UniProt protein use the low frequency minor allele as the reference. Finally, in those positions where RefSeq mRNA and UniProt protein represent the alternatives allele of GRCh38 (Figure 3D), the distribution is bimodal with high fractions of both low frequency (<5%) and high frequency (>70%) alternative alleles if the GRCh37 allele is different from that in GRCh38 (yellow), whereas alternative alleles predominate if GRCh37 is the same as GRCh38 (grey). Overall, these distributions show that a considerable fraction of the discordant variants between GRCh38, RefSeq mRNA, UniProt protein and GRCh37 can be classified into low (<5%) or high (>70%) frequency AAF groups.

**Table 1.** The numbers of concordant and discordant non-synonymous SNVs of 1K genomes data

GRCh37	RefSeq	UniProt	Occurrence
Ref	Ref	Ref	584 059
Alt	Ref	Ref	76
Ref	Alt	Ref	404
Alt	Alt	Ref	0
Ref	Ref	Alt	93
Alt	Ref	Alt	186
Ref	Alt	Alt	33
Alt	Alt	Alt	34

Each row represents a set of allele types of GRCh37 (previous reference genome), RefSeq and UniProt reference sequences and the occurrence of SNVs of the allele types. ‘Ref’ and ‘Alt’ indicate that the reference sequence of the column is the same as and different from the sequence of GRCh38, respectively.

Considering that the reference genome, RefSeq mRNA and UniProt have their own policies and strategies to include reference sequences, the concordance between the three sequences in 99.9% of the 1K genomes SNVs is quite high. The remaining differences are so small that a number of researchers of biology are unaware of them, but these differences may cause miscommunications between the researchers. Thus, decreasing the differences in the reference sequences will be valuable, and the AAF distributions of the discordant sites will be useful for this purpose. We will provide the full list of 1K genomes SNVs in the coding region and the allele representations of these SNVs in RefSeq mRNA and UniProt proteins upon request.

### Discordant variants with functional differences

We then examined how these discordances between the reference sequences impact functional studies of proteins. We found several variants in which UniProt represents a different sequence from that in GRCh38 and RefSeq, which leads to functional differences between the polymorphic sequences. One example is residue 389 of the β-1 adrenergic receptor (*ADRB1*), which is Gly based on the corresponding codon in GRCh38 and RefSeq (NM_000684.2), whereas it is Arg in UniProt (accession P08588) with an AAF of 30% (rs1801253) (32). *ADRB1* with Gly389 shows reduced binding to G proteins and reduced β-agonist-promoted contractility as compared to Arg389, while that with Arg389 shows a stronger β-blocker response than Gly389, indicating this polymorphism may be a therapeutic target in personalized medicine for heart failure (33).

As another example, the 30th residue of the proteinase-activated receptor 2 (*F2RL1*) gene is Asn in both the UniProt entry P55085 and RefSeq entry NM_005242.4, whose 30th codon is AAT, but the corresponding codon derived from GRCh38 is AGT, encoding Ser. This substitution is designated as rs616235 in dbSNP, with an AAF of 96.5%, indicating that Asn is predominant at this position. According to UniProt, Asn30 was experimentally shown to be N-glycosylated, which will not occur if the residue is Ser (34).

The functional effects of the amino acid changes are annotated in total 141 SNVs by using the following sources, (i) GWAS catalogue (17 SNVs) (35), (ii) ClinVar (111 SNVs) (36) and (iii) UniProt variant annotations (63 SNVs). The full list of discordant variants and functional annotations is included in the Supplementary Data.

### Discordant or rare reference variants affecting protein structures

We then examined how the discordance between different reference databases affects the amino acid sequences of those proteins with 3D structures registered in the PDB. Although PDB sequences are sometimes engineered from the UniProt sequences to enable structure determination or to test the effect of amino acid variation, we were interested in whether there are any protein structures that include a rare amino acid variant, which was not intentionally introduced but was used because other reference sequences, such as UniProt, represent it. We focused on the discordant variants between GRCh38, RefSeq and UniProt or known SNVs for which GRCh38 has the minor allele as the reference allele. In total, 168 discordant non-synonymous variants and four all-concordant variants with small reference allele frequencies < 5% are identified in the PDB structures. Some of these structures indicate that protein sequences including very rare variant alleles are used for structure analyses presumably because the reference databases represented the rare alleles.

**Figure 4.**
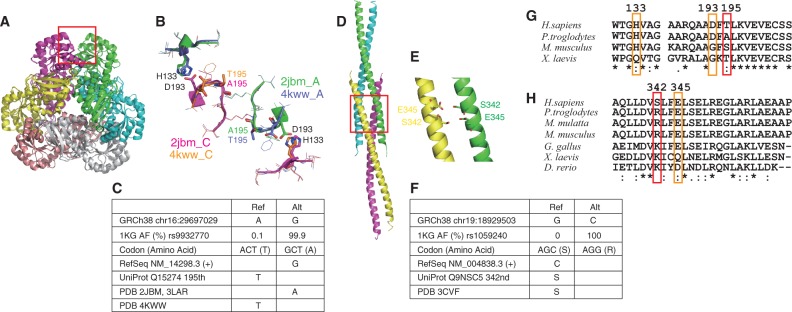
Protein 3D structures including rare variants. (A) Superimposition of two quinolic acid phosphoribosyl transferase (QPTR) hexamers. (B) Dimer–dimer interface of QPTR surrounding the variant residues A195T. Chain As of PDB entries 2JBM and 2KWW are green and purple, respectively and their chain Cs are magenta and orange, respectively. (C) A rare variant in QPTR. (D) Structure of HOMER protein homologue 3 (HOMER3) coiled-coil domains and (E) close-up view with the S342 and E345 residues. (F) A rare variant in HOMER3. (G, H) Sequence comparisons of (G) QPTR and (H) HOMER3.

One of the cases is human quinolinic acid phosphoribosyltransferase (*QPRT*, RefSeq NM_014298.3, UniProt Q15274, Figure 4A and B). The chromosome 16 position 29697029 is A in GRCh38, for which an alternative allele G is observed with an AAF of 0.999 in the 1K genomes (rs9932770, Figure 4C). This variant changes the 195^th^ amino acid from Thr (reference, rare) to Ala (alternative, major). RefSeq (NM_014298.3, which is now updated to NM_014298.4 without changing its CDS sequence) and UniProt (Q15274) have the alternative and reference alleles of this variant, respectively, as their references. *QPRT* functions as a hexameric complex, with three-fold rotational symmetry consisting of three dimers, each formed with two-fold rotational symmetry (37). The 195^th^ amino acid is located at the interface between the neighbouring dimers, and the 3D structures with both Ala [PDB 2JBM (37) and 3LAR (38)) and Thr (4KWW (39)] are available. The change of this amino acid from the major Ala to the rare Thr introduces a small change in the dimer–dimer interface conformation. If the 195th residue is Ala, then its side chain does not form an intra-molecular hydrogen bond and Asp193 can make a side-chain-side-chain hydrogen bond with His133 of the neighbouring chain across the dimer–dimer interface. However, if this residue is Thr, then its side chain oxygen hydrogen-bonds to the side-chain of Asp193, and the hydrogen bond between Asp193 and His133 across the interface is disrupted (Figure 4A and B). As this dimer–dimer interface undergoes significant conformational changes upon substrate and product binding (38), this variant may affect the function of *QPRT*. These three residues, Ala195, Asp193 and His133, are conserved in primates, but not in mammals or vertebrates, indicating the importance of this interface in primates (Figure 4G).

Another example is the *HOMER3* protein, a member of the HOMER family of post-synaptic density (PSD) scaffolding proteins (Figure 4D and E). *HOMER3* forms a homo-tetramer via interactions between the carboxyl terminal coiled-coil domains of the subunits, which assemble numerous channels and other scaffolding proteins in the network structure within the PSD. The coiled-coil region of *HOMER3* was solved in the tetrameric complex, and revealed the tail-to-tail interaction of two coiled-coil dimers (40). In the coding region of this protein, chromosome 19 position 18929503 is G in GRCh38, for which an alternative allele C is observed with an AAF of 100% in the 1K genomes (Figure 4F). This variant changes the 342nd amino acid from Ser (reference, rare) to Arg (alternative, major). Both RefSeq (NM_004838.3) and UniProt (Q9NSC5) have Ser at this position, consistent with GRCh38, but a rare allele. The crystal structure of *HOMER3* (PDB 3CVF) has Ser at this position, although all of the samples in the 1K genomes have Arg at this position (40). This Ser faces Glu345 in a neighbouring chain of the other dimer (Figure 4E). If it is Arg, then a salt bridge can be formed between the two dimers, possibly enhancing their interaction. Arg is observed in mammals, and positively charged residues occur in vertebrates at this position, suggesting the importance of the electrostatic interaction (Figure 4H). As a stable tetrameric structure is crucial for the scaffolding function of HOMER proteins and the correct formation of the PSD, this amino acid mutation may have not only structural but also biological influence.

These results indicate that if the reference databases represent the minor allele of a variant site, the structural and functional analyses are sometimes biased using the protein sequence including the amino acid corresponding to the minor or rare allele of the variant.

### Role of RNA-editing in the differences between genome and mRNA sequences

We next considered the possibility that the discordance between the GRCh38 and RefSeq mRNA sequences originated from biological differences between genome and transcript due to post-transcriptional modifications, such as RNA-editing. RNA-editing is a process where, in the context of a nearly palindromic sequence within an mRNA or its precursor molecule, an adenine base is deaminated to yield an inosine, which performs a biologically equivalent function to a guanine, in terms of reverse transcription and translation. Although RNA-editing is rare in CDS because it requires palindromic sequences, there are 54 transcripts for 23 genes with the annotation ‘undergo RNA-editing’ by RefSeq in our data set. Among three of these genes, *NARF*, *GRIA3* and *GRIA4*, we found five discordant variants changing the mRNA base from A to G, which were previously reported as RNA-editing sites. These variants were not reported in either the 1K genomes study or the dbSNP, indicating that these changes are not genetically rooted.

**Figure 5.**
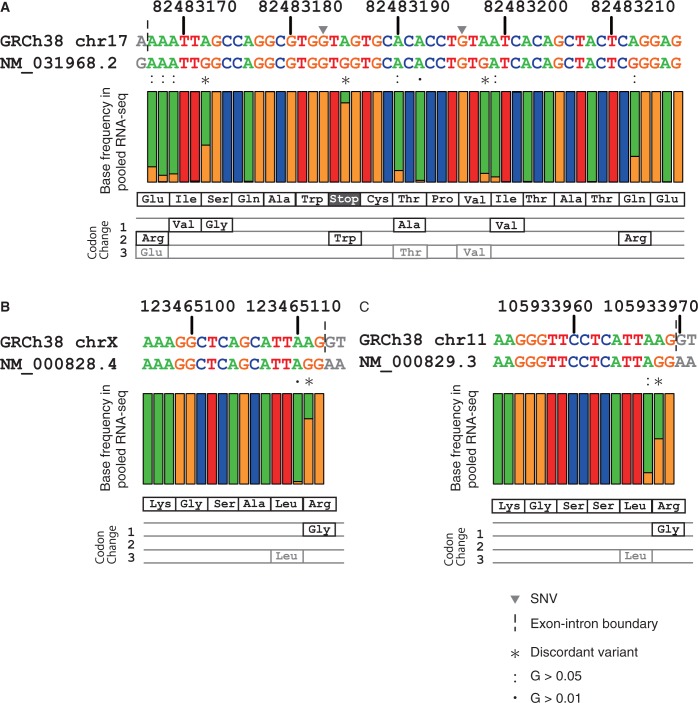
Base frequency in pooled RNA-seq data of the GEUVADIS project for regions surrounding discordant variants for (A) NM_031968.2 (NARF), (B) NM_000828.4 (GRIA3) and NM_000829.3 (GRIA4). A, T, G and C bases are coloured green, red, orange and blue, respectively, in the nucleotide sequences and bar plots. The bar plots indicate the fraction of each base occurring in the pooled RNA-seq data of 462 individuals from the GEUVADIS project. The amino acid sequences for these regions and the amino acid changes that occur due to possible A-to-G RNA-editing events are shown below the bar plot, with synonymous changes shown in grey. Grey inverted triangles on the DNA sequences represent two SNVs within these regions, which are both rare (0.02% in 1,000 genomes phase 3) and are G-to-A changes. The vertical dashed lines indicate exon–intron boundaries, and the grey letters indicate the intronic bases in the DNA sequences and the RNA bases from neighbouring exons of the transcript sequences. Asterisks indicate the discordant variants, and colons and dots indicate possible RNA-editing with G-base fractions of 0.05 and 0.01, respectively.

To examine the extent of RNA-editing within these positions in the human population, we used RNA-seq data from the GEUVADIS project for 462 individuals in the 1K genomes. Figure 5 shows the GRCh38 and RefSeq mRNA sequences around the RNA-editing sites, with the percentages of base occurrence by pooled RNA-seq data. We identified three previously reported RNA-editing sites in exon 8 of the human nuclear prelamin A recognition factor (*NARF*) gene (41). This exon originated from an intronic *Alu* element that evolved to have functional splice sites, in a process called exonization, and only isoform 2 of *NARF* (NM_031968.2) includes this exon. The bases at three positions, 82483185, 82483198 and 82483212, of chromosome 17 are As in GRCh38, but the corresponding ones in RefSeq NM_031968.2 are Gs. The variants corresponding to these substitutions were not reported in either the 1K genomes, the ESP6500 studies or the dbSNP, but the RNA-seq data show that this position consists of a mixture of A and G, together with some additional A to G substitutions within this exon (Figure 5A). In particular, the change of the 82483185th base is crucial for the expression of this isoform, because it converts a premature stop codon (TAG) at the 263rd position to tryptophan (TGG). The extent of RNA-editing correlated with the functional importance of the site, because the stop-loss editing site at the 82483185th position occurs most frequently. These results indicate that the three base differences between GRCh38 and RefSeq NM_031968.2 are the result of an RNA-editing process, but these discordant positions are not the only sites undergoing RNA-editing.

The two other examples occur in two subtypes of glutamic acid receptor ionotropic AMPA (GRIA), the *GRIA3* (NM_000828.4) and *GRIA4* (NM_000829.3) genes (Figure 5B and C). The four GRIA genes (*GRIA1* to *GRIA4*) have the same AGA codon (encoding Arg) at the junction of two exons, where the AG dinucleotides of this codon reside in the 5′ side exon (42, 43). The change of the first A base of this codon to G leads to the Arg to Gly amino acid change, which reportedly occurs in *GRIA2*, *GRIA3* and *GRIA4*, but not in *GRIA1* (42). This base is A in GRCh38 for all four GRIAs, whereas it is G (e.g. edited) for *GRIA3* and *GRIA4* but A (e.g. non-edited) for *GRIA1* and *GRIA2* in the RefSeq mRNAs, indicating that the RefSeq mRNA sequences of *GRIA1*, *GRIA3* and *GRIA4* are consistent with the annotated RNA-editing statuses, but that of *GRIA2* is not. Consistent with the annotations, at least half of the aligned bases in the pooled RNA-seq data are G for this position in *GRIA3* and *GRIA4* (Figure 5B and C), but all of the aligned bases are A in *GRIA1* (data not shown) and no reads were mapped on this region of *GRIA2*.

Although this survey was restricted to the RNA-seq data from lymphoblastoid cells (28), we found some of the discordant bases between GRCh38 and RefSeq mRNA that have annotations of RNA-editing, but did not find any evidence that other discordant bases are derived from RNA-editing or other post-transcriptional modifications. Thus, we conclude that post-transcriptional modifications play a limited role in the differences between the genomic (GRCh38) and transcript (RefSeq mRNA) reference sequences.

### Classification differences between UCSC and CCDS

As shown in Supplementary Table S1, we found many classification differences between the three gene loci annotations by UCSC, CCDS and BLAT. We discuss the causes of these differences in the Supplementary Discussion. In many cases, the insertion of a micro-exon within the intron can erase apparent base mismatches on the exon–intron boundary neighbouring it (Supplementary Figure S1). In addition, SNVs with large AAFs can hinder the accurate determination of exon-intron boundaries (Supplementary Figure S2). Long introns spanning ∼1 megabases can also be misidentified (Supplementary Figure S3). Overall, the annotation by the CCDS collaboration produces more canonical splicing codes and better match the GEUVADIS RNA-seq reads than that by the UCSC. These differences can be derived from the difference in their methodologies; the UCSC annotation is primarily based on BLAT-based automatic alignment, while the CCDS annotation is produced by the whole-genome annotation pipelines with extensive manual review. These results indicate that the accurate identification of coding regions requires extensive manual curation, especially where the reference genome and mRNA sequences represent different alleles.

### Missing bases in the reference genome (class 3)

We then examined the difference between the GRCh38 and Class 3 RefSeq mRNA sequences, which include missing sequences in the CDS; i.e. transcript bases that are not found in the GRCh38 reference genome. By classifying the 41 Class 3 mRNAs by gene names and genomic positions, we obtained the missing sequences for 24 genes (Table 2). Most of the missing sequences are short (<10) although some genes, such as MUC19, MUC2 and FCGBP, have long (>100) missing sequences.

**Table 2.** Missing gene segments in GRCh38

GENE	Description	#1	Chr	Strand	Positions	#2	mRNA
ABO	Abo blood group (transferase a, alpha 1-3-n-acetylgalactosaminyltransferase; transferase b, alpha 1-3-galactosyltransferase) (abo)	1	chr9	−	133257521:133257521	G	NM_020469.2
THSD7B	Thrombospondin, type i, domain containing 7b (thsd7b)	1	chr2	+	137451025:137563217	T	NM_001080427.1
TMEM247	Transmembrane protein 247 (tmem247)	1	chr2	+	46484425:46484425	A	NM_001145051.2
IFNL4	Interferon, lambda 4 (gene/pseudogene) (ifnl4)	1	chr19	−	39248513:39248515	C	NM_001276254.2
PKD1L2	Polycystic kidney disease 1-like 2 (pkd1l2)	1	chr16	−	81127878:81127878	G	NM_001278425.1, NM_052892.3
NR1H2	Nuclear receptor subfamily 1, group h, member 2 (nr1h2)	3	chr19	+	50378567:50378567	ACA	NM_007121.5, NM_001256647.1
ALMS1	Alstrom syndrome 1 (alms1)	3	chr2	+	73385903:73385903	GGA	NM_015120.4
BCL6B	B-cell cll/lymphoma 6, member b (bcl6b)	3	chr17	+	7024730:7024730	CAG	NM_181844.3
HTT	Huntingtin (htt)	6	chr4	+	3074935:3074935	GCAGCA	NM_002111.7
LACTBL1	Lactamase, beta-like 1 (lactbl1)	7	chr1	−	22965430:-	ATGAAGA	NM_001289974.1
NPIPB3	Nuclear pore complex interacting protein family, member b3 (npipb3)	11	chr16	−	21404379:21404384, 21404506:21404509	AGCTC, GCTCAC	NM_130464.2
IGFBP2	Insulin-like growth factor binding protein 2, 36kda (igfbp2)	9	chr2	+	216633587:216633587	CGCTGCTGC	NM_000597.2
MOB2	Mob kinase activator 2 (mob2)	16	chr11	−	1480886:-	ATGGACTGGCTC	NM_053005.5
						ATGG	
MROH8	Maestro heat-like repeat family member 8 (mroh8)	29	chr20	−	37179390:37179390	AAGAGTGCCGGC	NM_152503.5, NM_213631.2, NM_213632.2
						CGCGGGGCCCTGTCTAT	
RYBP	Ring1 and yy1 binding protein (rybp)	30	chr3	−	72446623:-	ATGACCATGGGC	NM_012234.6
						GACAAGAAGAGCCCGACC	
NRG1	Neuregulin 1 (nrg1)hrg-beta1	35	chr8	+	−:32595825	–	NM_001159995.1, NM_001159999.1
SHANK3	Sh3 and multiple ankyrin repeat domains 3 (shank3)	42	chr22	+	50695048:50697563	–	NM_033517.1
CASP8AP2	Caspase 8 associated protein 2 (casp8ap2)	48	chr6	+	89871390:89873802	–	NM_012115.3, NM_001137667.1, NM_001137668.1
FAM101B	Family with sequence similarity 101, member b (fam101b)	209	chr17	−	445940:-	–	NM_182705.2
NBPF1	Neuroblastoma breakpoint family, member 1 (nbpf1)	220	chr1	−	–	–	NM_017940.4
SAMD1	Sterile alpha motif domain containing 1 (samd1)	318	chr19	−	14090084:14090084	–	NM_138352.1
MUC19	Mucin 19, oligomeric (muc19)	487	chr12	+	–	–	NM_173600.2
MUC2	Mucin 2, oligomeric mucus/gel-forming (muc2)	3153	chr11	+	–	–	NM_002457.3
FCGBP	Fc fragment of igg binding protein (fcgbp)	3603	chr19	−	–	–	NM_003890.2

#1: Number of missing bases.

#2: Missing mRNA base.

A typical example of a gene with a missing sequence is the *ABO* gene. The *ABO* gene is one of the genes responsible for determining the ABO blood groups (44), and the SNP rs8176719 within it describes the polymorphism of the phenotype. The alternative allele of this locus has an insertion of a G base, as compared with the reference allele. The coding sequence of the alternative allele with the inserted G base is translated into the intact ABO protein, which leads to the glycosylation of red blood cells in the A and B blood groups, whereas the coding sequence of the reference allele with the deletion of the G base causes a frame-shift mutation, which leads to the ABO protein without catalytic function, resulting in the O blood group phenotype. The reference genome has the reference allele without the G base because it represents the majority in the human population, although it is not functional. Both of the RefSeq mRNA and UniProt reference sequences are consistent with the alternative allele, which produces the functional protein. In this case, GRCh38 and RefSeq or UniProt are different because the majority in the population and the functionality do not coincide with each other.

Several cases of missing sequences are derived from short tandem repeats in the CDS. The positions where the number of mRNA sequence bases that are missing from the reference genome is a multiple of three are caused by amino acid insertion/deletion polymorphisms. For example, some proteins include a stretch of a repeated single amino acid, which is translated from a repeat of the same codons. The number of such repeated codons can vary among individuals, which leads to the discordance between the reference databases. For example, the *ALMS1* mRNA in RefSeq (NM_015120.4) has a tandem repeat of thirteen GAG codons, whereas the reference genome has only twelve GAG codons at the locus (45). This GAG repeat and the downstream one GAA and three GAG codons are translated into seventeen (RefSeq mRNA) and sixteen (based on the reference genome) Glu amino acids in the protein sequence. The UniProt *ALMS1* protein (Q8TCU4) has 16 glutamic acids in this repeat; i.e. it is consistent with the reference genome. The missing segments of three bases are also observed in the *NR1H2*, *BCL6B*, *HTT* and *IGFBP2* genes. A GC-rich region of 318 bases in the *SAMD1* gene is another case of a repetitive missing sequence in the reference genome. Missing sequences longer than three bases are usually complex, and may indicate that the region of the reference genome has not been determined accurately. The genomic sequences including these missing gene sequences are expected to be determined in the future.

## Conclusion

The DNA and mRNA reference databases have been compared previously, but to the best of our knowledge, this is the first attempt to incorporate protein-level evidence in comparison to the genomic and transcriptomic sequences. Protein-level information, which includes residue-level annotations of structural and functional sites, is necessary to assess the impact of an SNV on protein structure and function (46, 47). However, if the sequences of the same gene are different between separate databases, then automatic mapping of the variants on the genomic coordinates to the protein amino acid substitutions will be hindered between databases. By comparing the reference sequences of DNA, mRNA and protein, we observed a significantly high-level of consistency between them. We also observed that the differences between RefSeq mRNA and GRCh38 originated from minor reference alleles in GRCh38, which is expected from the vast majority of rare variants in current population genomics studies (24). In addition, many minor alleles still remain that are used as references by both GRCh38 and RefSeq mRNA. A comparison between GRCh37 and GRCh38 revealed that many of the inconsistent sequences observed between GRCh37 and RefSeq mRNA have been corrected between these versions of the genome. As the allele frequency data have become saturated for polymorphic sites, it will be beneficial for future genome analyses if the reference sequences are unified to represent the major alleles at as many sites as possible.

## Supplementary data

Supplementary data are available at *Database* Online.

Supplementary Data
