# Supplementary material for: Discrepancies between human DNA, mRNA and protein reference sequences and their relation to single nucleotide variants in the human population
Source: Database (Oxford). 2016 Sep 1;2016:baw124. doi: 10.1093/database/baw124 (PMC5009343; doi:10.1093/database/baw124)
Supplement: Supplementary Data [file supp_2016_baw124_index.html]

Discrepancies between human DNA, mRNA and protein reference sequences and their relation to single nucleotide variants in the human population — Supplementary Data 

# Discrepancies between human DNA, mRNA and protein reference sequences and their relation to single nucleotide variants in the human population

## Supplementary Data

files

- Supplementary Data - xlsx file
- Supplementary Data - pdf file
