# Supplementary material for: Discrepancies between human DNA, mRNA and protein reference sequences and their relation to single nucleotide variants in the human population
Source: Database (Oxford). 2016 Sep 1;2016:baw124. doi: 10.1093/database/baw124 (PMC5009343; doi:10.1093/database/baw124)
Supplement: Supplementary Data [file supp_baw124_SupplementaryMaterial.pdf]

Supplementary Discussion

As shown in Supplementary Table 1, we found many classification differences between the three gene loci annotations; i.e., UCSC, CCDS and BLAT. For example, 49 mRNAs were class 2 (substitution) by UCSC, but were class 1 (exactly match) by CCDS and BLAT, whereas 63 mRNAs were class 1 by UCSC, but were class 2 by CCDS. As the UCSC and CCDS gene annotations are both widely used, it is valuable to study the detailed differences between them in the annotations of these mRNAs.

Among the transcripts classified as class 2 (substitution) by UCSC but as class 1 (exactly match) by CCDS and BLAT, the mismatches between GRCh38 and RefSeq mRNA are frequently derived from exon-intron boundaries, where a micro-exon is included by CCDS but not by UCSC. For these cases, we found that the UCSC annotations are likely to be inaccurate, as described below. For example, the alignment of the CDS of the kinase suppressor of Ras 1 (*KSR1*) gene (NM_014238.1) to GRCh38 by UCSC is separated by an intron spanning the 27583108th to 27588471st bases of chromosome 17, but this alignment yields two mismatching bases between

GRCh38 and RefSeq mRNA in the succeeding exon (Supplementary Fig. S1A). However, this intron is split into two, and expanded by two bases on the 5' side and three bases on the 3' side, and includes a micro-exon consisting of five bases from the 27585657th to 27585661st bases of chromosome 17. The two bases (TT) of mRNA that are mismatched in the UCSC alignment match two bases in the micro-exon, thereby eliminating mismatches. The two split introns generated by the CCDS alignment, but not the intron generated by the UCSC alignment, have the canonical splicing code; i.e., starting with a GT dinucleotide on the 5' side and ending with an AG dinucleotide on the 3' side, which indicates that the introduction of the micro-exon is consistent with the RNA-splicing machinery. These regions were devoid of SNVs in the genomic sequence of the 1K genomes samples, and the RNA-seq reads of the GEUVADIS samples are consistent with the sequence of RefSeq NM_014238.1 (Supplementary Fig. S1A). As the DNA-sequencing and RNA-sequencing results support the GRCh38 and mRNA sequences, respectively, the alignment generated by CCDS is more appropriate than that by UCSC for this mRNA. Among the mRNAs that were classified as class 1 by CCDS but as class 2 or 3 by UCSC, the majority of the mRNA bases that are

mismatching or missing are located near exon-intron boundaries and could be matched to the GRCh38 sequence, if a micro-exon is inserted in the intron neighboring them and the exon-intron boundaries are adjusted by a few bases (Supplementary Fig. S1A, C-AS). The coverage of pooled RNA-seq reads ranged from several hundreds of thousands to none for the mismatching bases between RefSeq mRNA and GRCh38, according to the UCSC exon annotations (Supplementary Fig. S1). The reads covering the mismatching bases, if any, exclusively supported the RefSeq mRNA sequences and the CCDS exons, but not the UCSC exons (Supplementary Fig. S1A, C-AH). In some cases, the coverage for micro-exons decreased as compared with the flanking regions of the mRNA, suggesting that they are alternatively-spliced micro-exons (Supplementary Fig. S1 U-AB). An exceptional case is the ArfGAP with GTPase domain, ankyrin repeat and PH domain 4 (*AGAP4*) gene, whose second exon is assigned to different regions by UCSC and CCDS, which differ from each other by one base. No SNVs were reported within these alternative two exons, and the pooled RNA-seq data show that both of these exons are expressed, yielding heterogeneous transcripts (Supplementary Fig. S1B).

Conversely, there were 49 transcripts that were classified as class 2 (substitutions) by CCDS but class 1 (exactly matched) by UCSC. Among them, 27 were mapped by UCSC on the alternative scaffolds, which were not considered by CCDS. The alternative scaffolds are parts of the human genome sequence that include polymorphic changes as compared with the reference chromosome sequences. Thus, the differences in the classification of these mRNAs between UCSC and CCDS are derived from genomic differences, and thereby both the UCSC and CCDS annotations can be correct. However, the differences in 20 out of the remaining 22 transcripts were explained by grouping them into six SNVs with large AAFs (Supplementary Fig. S2). For instance, the outer dense fiber of sperm tails 2 (*ODF2*) gene, RefSeq NM_007005.4, is aligned to chromosome 9 perfectly by the UCSC alignment but with one base mismatch at an exon-intron boundary by the CCDS alignment, in which the exon-intron boundary is shifted to the 3' end by one base from that of the UCSC alignment (Supplementary Fig. S2A). In the CCDS alignment, the 45th base G of the mRNA mismatches the A base at chromosome 9 position 79542835. This substitution is annotated as rs128473041, with the AAF of the G base being 99%, indicating that

GRCh38 selects the rare allele. If the reference genome sequence is changed to the major allele, then the alignment by CCDS produces a perfect match of the mRNA and the genome sequence. The alignment by CCDS makes a canonical splicing code in the intron between these exons, whereas that of UCSC does not. In the other five cases for which CCDS assigned regions on canonical chromosomes, SNVs with large AAFs were found that will make perfect matches between GRCh38 and RefSeq mRNA by the CCDS alignment, if GRCh38 selected the alternative alleles as the references (Supplementary Fig. S2 B-F). The allele information in the remaining two transcripts was not found in either the 1K genomes or the RNA-seq results from the GEUVADIS data, and thereby these discordances have not been solved with the current data. The alignment by CCDS is more likely to be accurate, because it takes the minor allele of the GRCh38 into account, whereas UCSC aligned the mRNA sequence to the GRCh38 as-is. These cases highlight the fact that an SNV with a large AAF presumably hampers the correct determination of exon-intron boundaries by automatic methods.

We found three mRNAs that were classified as class 1 by CCDS and class 3 by UCSC and BLAT: roundabout, axon guidance receptor, homolog 2 (Drosophila)

(*ROBO2*) transcript variant 1 (NM_001128929.3), neuregulin 1 (*NRG1*), transcript variant HRG-β 1d (NM_001160001.1), and Kv channel interacting protein 4 (*KCNIP4*), transcript variant 1 (NM_025221.5). For these mRNAs, we found that the first exon is about 1Mb upstream of the second exon in the CCDS alignment, whereas this exon is missing in the UCSC alignment (Supplementary Fig. S3). As a result, we employed the CCDS exon definitions for those transcripts that are classified as class 1 by CCDS.

These results indicate that the accurate identification of coding regions requires extensive manual curation. In many cases, the insertion of a micro-exon within the intron can erase apparent base mismatches on the neighboring exon-intron boundary. As an SNV with a large AAF can hinder the accurate determination of exon-intron boundaries, the reference genome is required to represent the major allele in as many variant positions as possible, for the accurate reconstruction of splicing isoforms in RNA-seq.

Supplementary Table S1.

UCSC Class	CCDS Class 1	2	3	4
1	33451/4/77/0	49/0/0/0	7/0/0/0	2587/1/9/0
2	41/1/21/0	8/1563/41/0	5/0/0/0	16/126/12/0
3	11/0/9/0	0/1/1/0	3/0/7/0	9/7/25/0
4	0/0/0/0	0/0/0/0	0/0/0/0	0/0/2/1

Supplementary Table S1. Numbers of RefSeq mRNAs classified by the comparison of coding sequence with GRCh38, according to UCSC (row), CCDS (column) and BLAT. The figures separated by slashes indicate the number of mRNAs classified by BLAT as Class 1, 2, 3 and 4, from left to right.

Supplementary Figure S1.

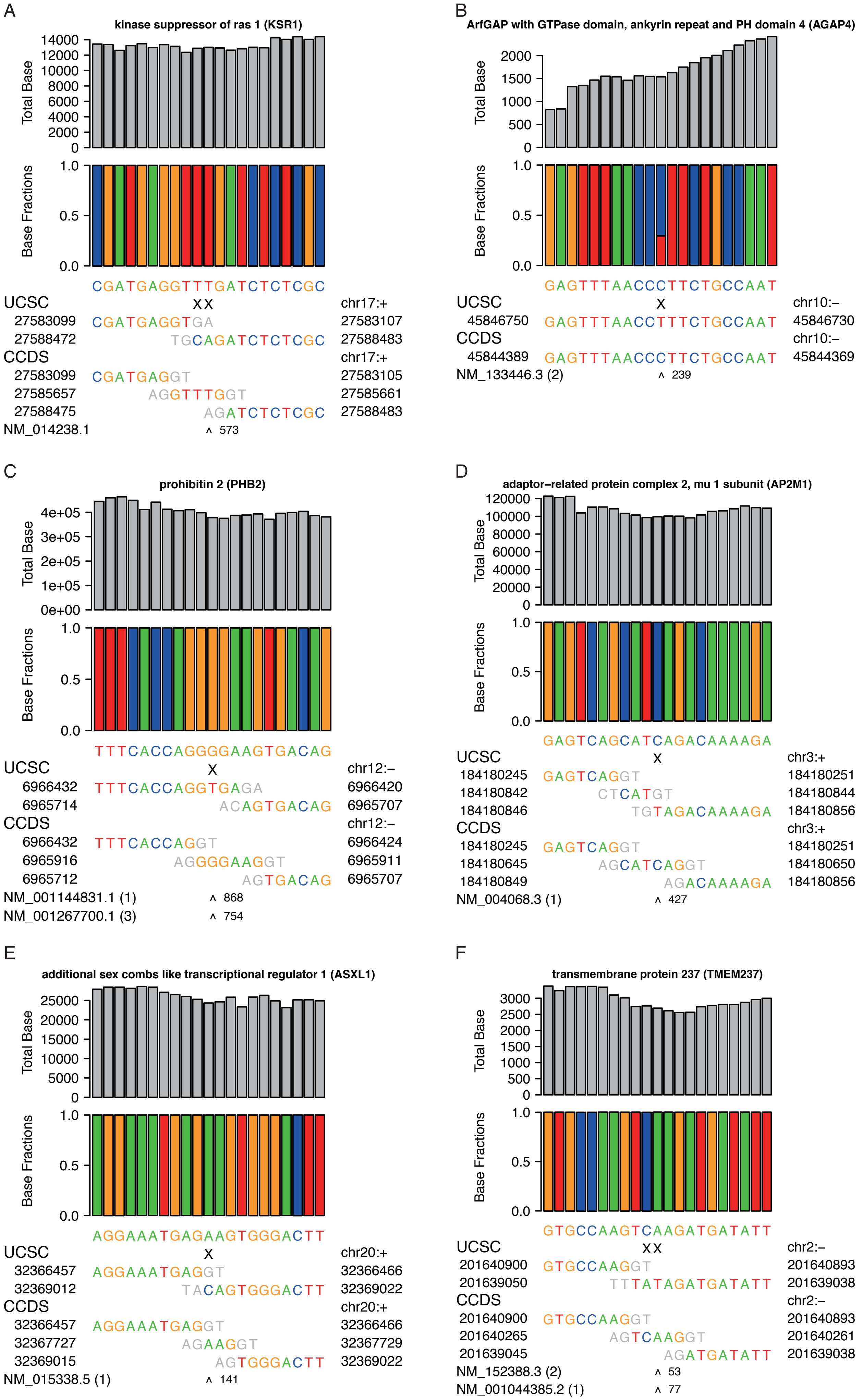

Supplementary Figure S1 (continued).

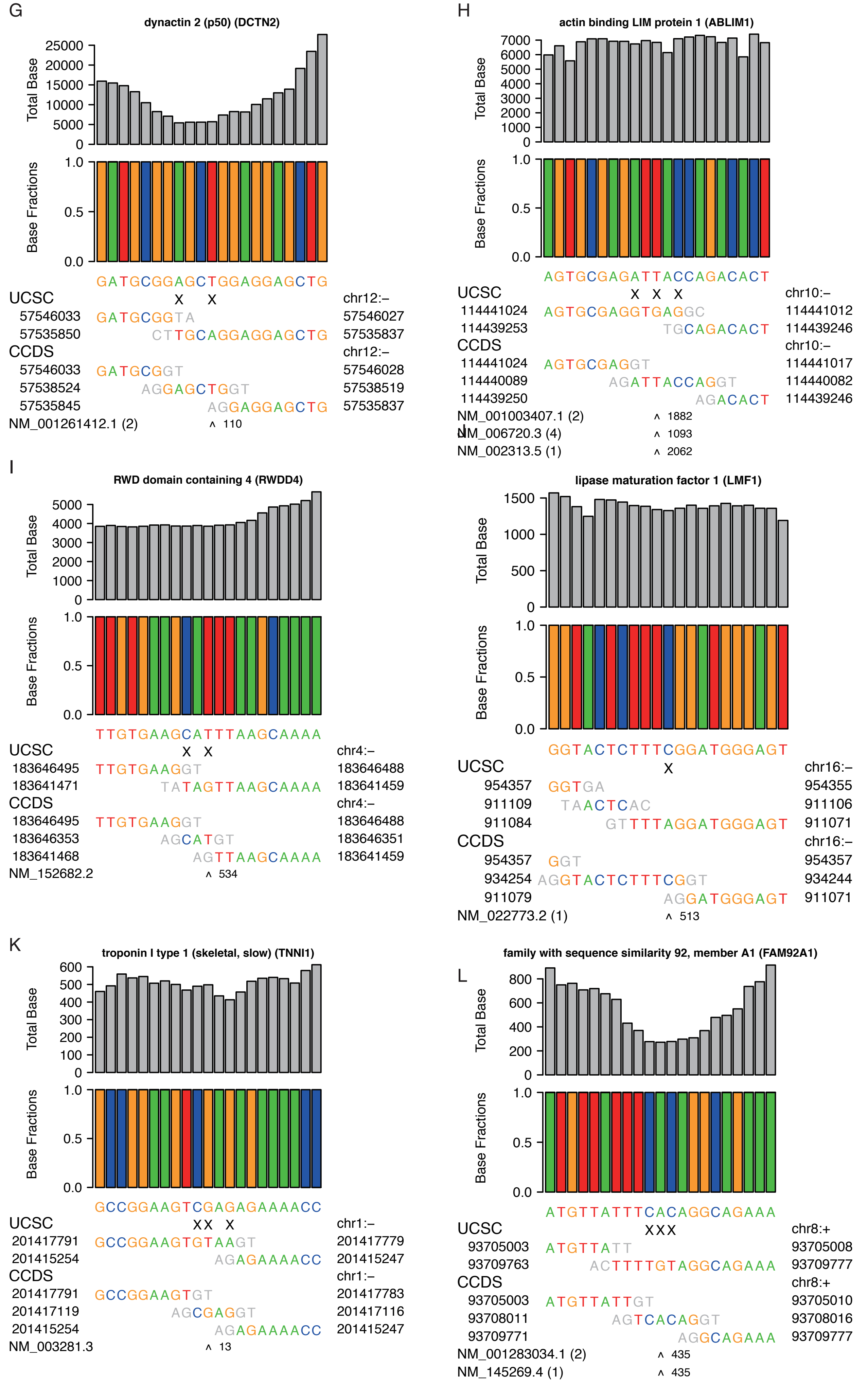

Supplementary Figure S1 (continued).

M

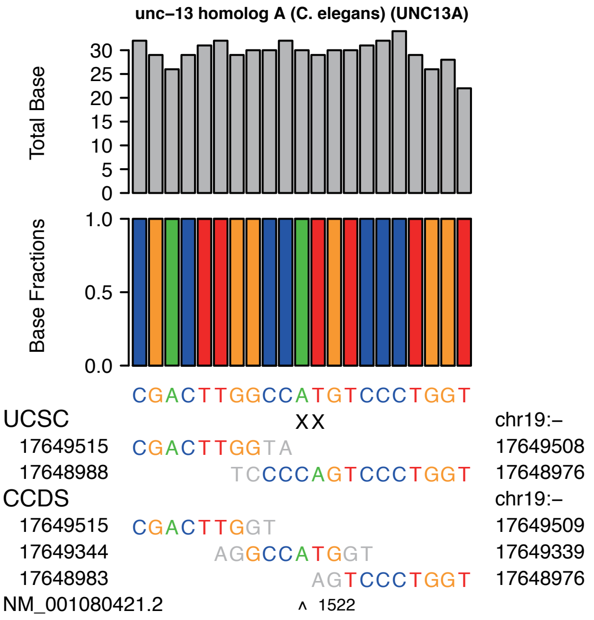

N

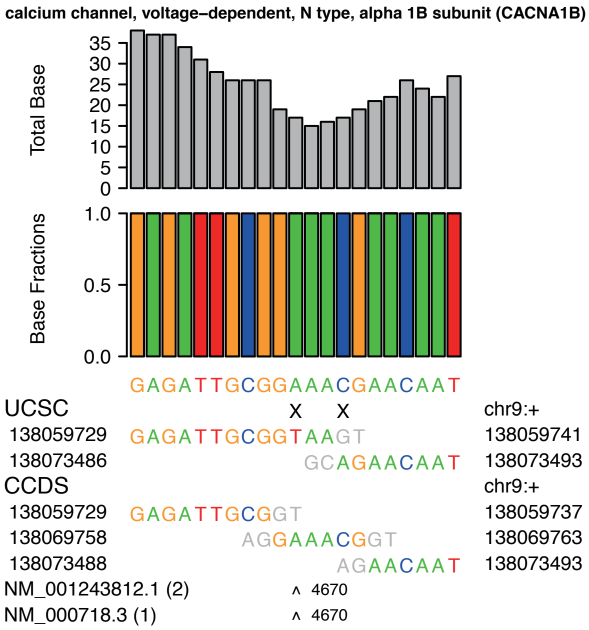

O

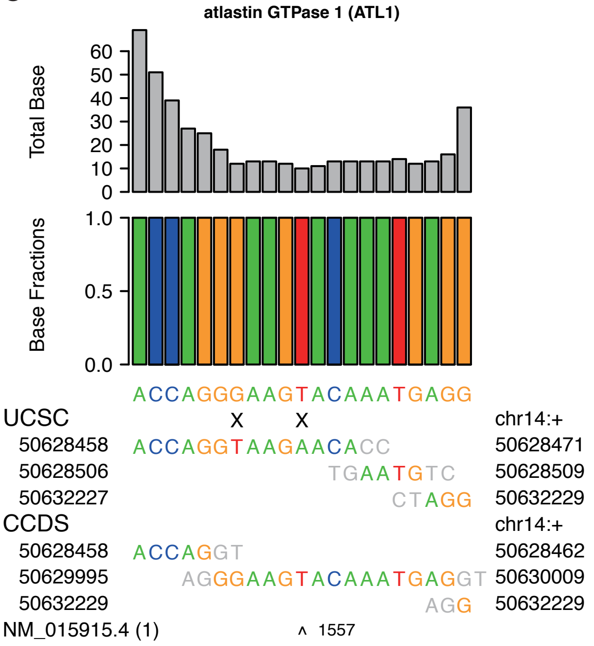

P

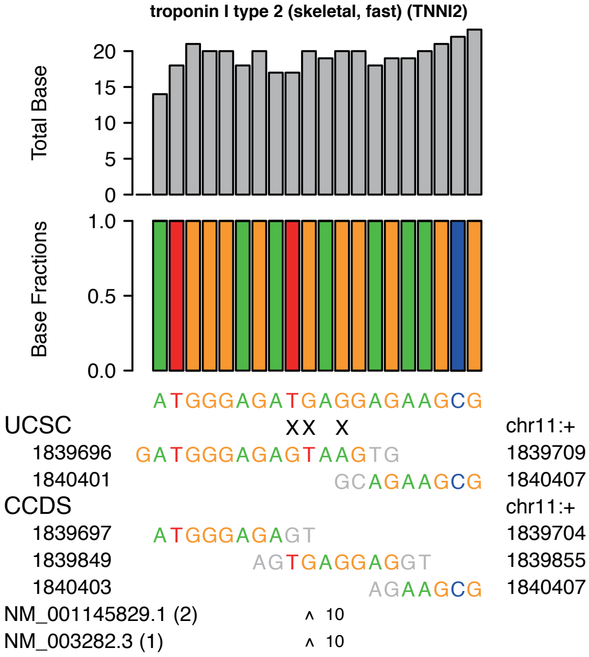

Q

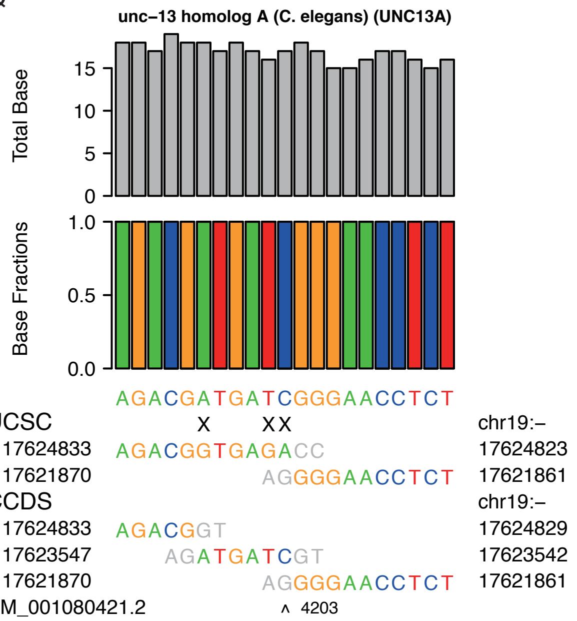

R

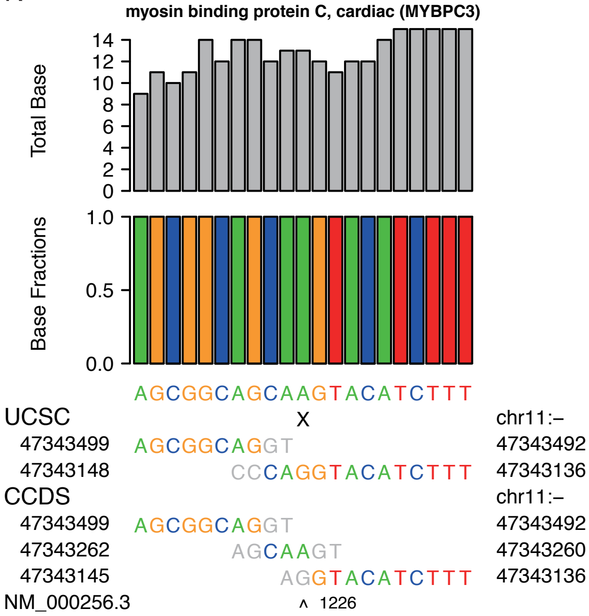

Supplementary Figure S1 (continued).

S

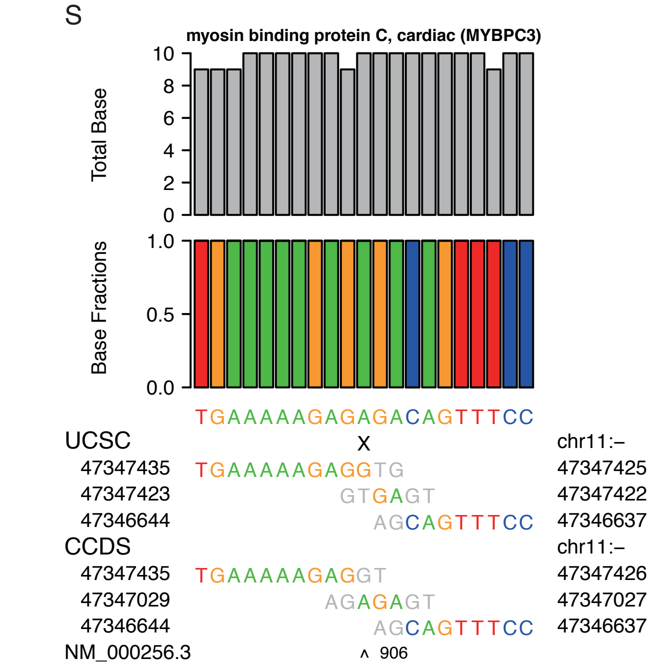

T

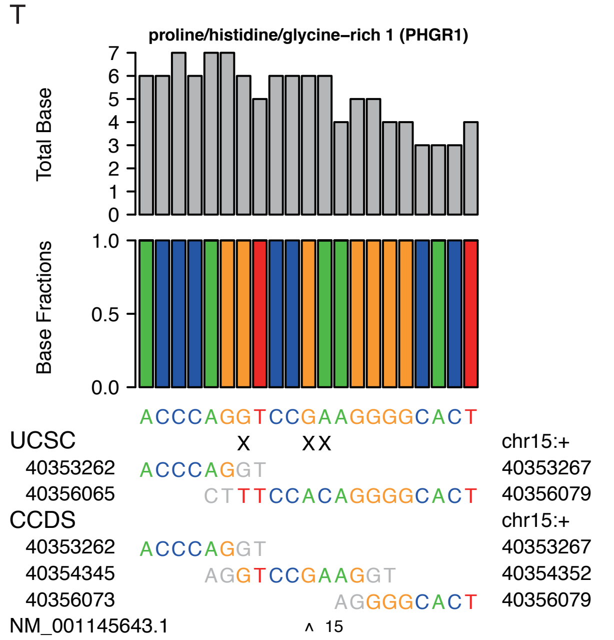

U

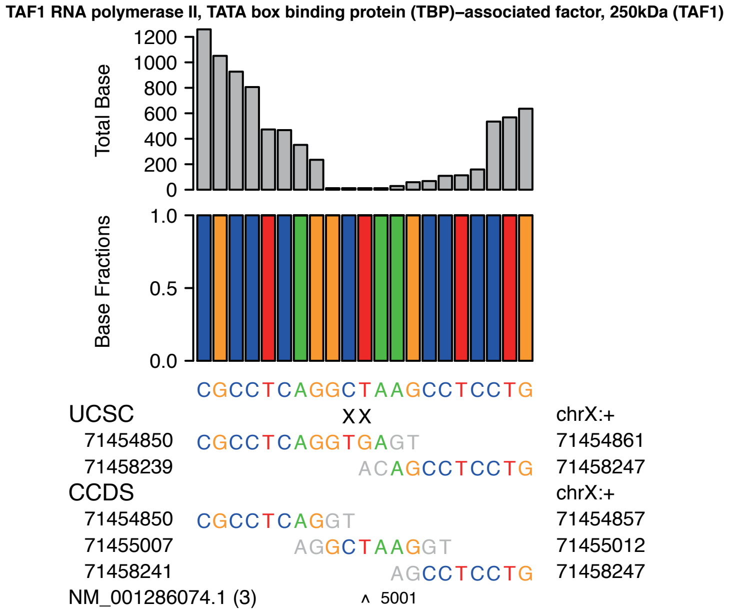

V

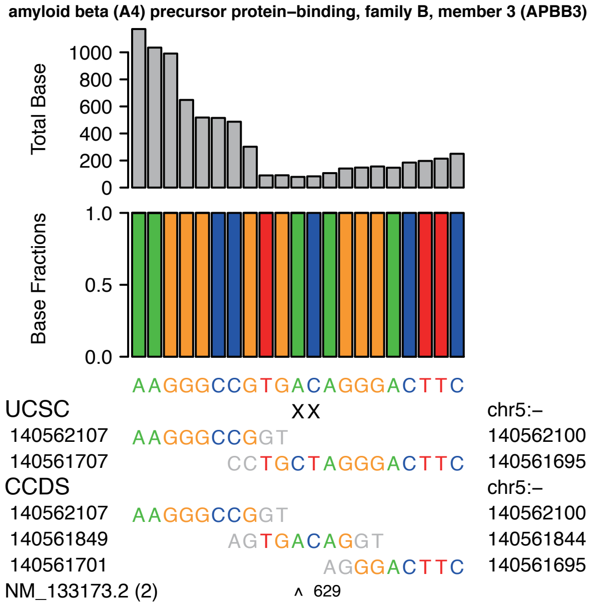

W

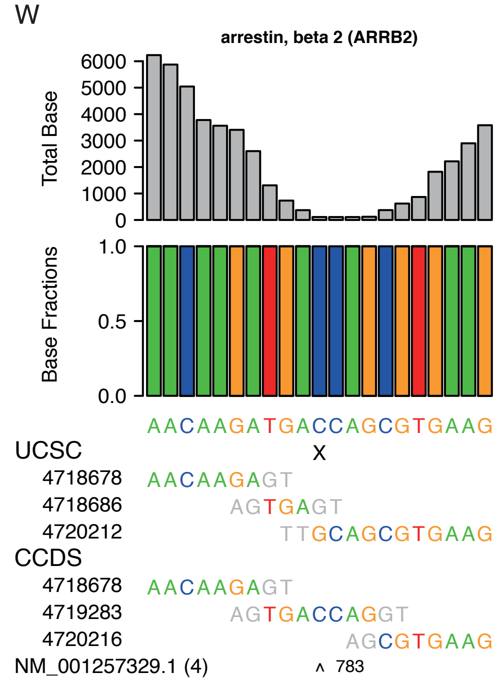

X

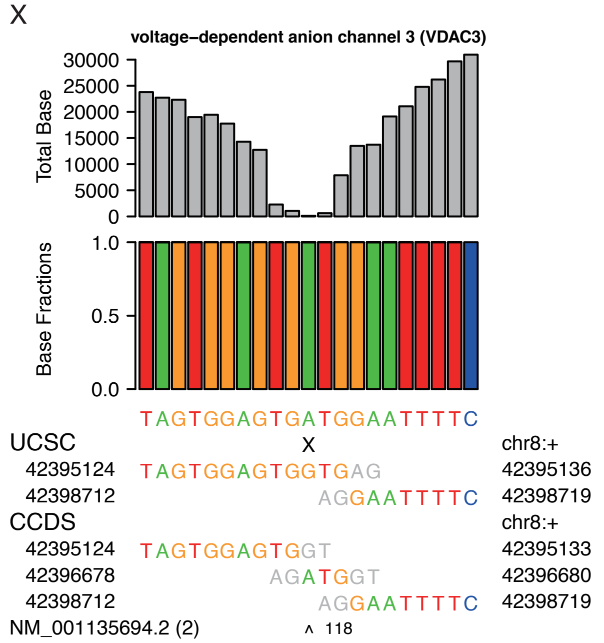

Supplementary Figure S1 (continued).

Y

C–type lectin domain family 16, member A (CLEC16A)

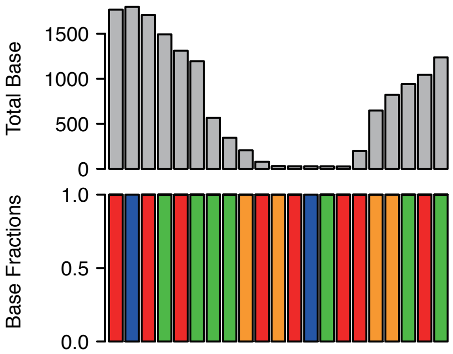

TCTATAAAGTGTCATTGGATA
UCSC X X chr16:+
10971222 TCTATAAAGGT 10971230
10972932 TTTCTCAGTGGATA 10972943
CCDS chr16:+
10971222 TCTATAAAGGT 10971230
10972554 AGTGTCATGT 10972559
10972938 AGTGGATA 10972943
NM_015226.2 (1) ^ 600

Z

NSFL1 (p97) cofactor (p47) (NSFL1C)

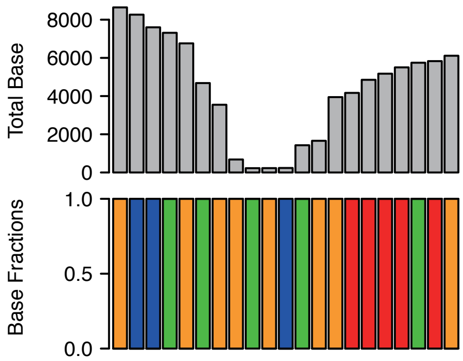

GCCAGAGGAGCAGGTTTTATG
UCSC X X chr20:–
1458206 GCCAGAGGTGAGT 1458196
1455134 ACAGGTTTTATG 1455125
CCDS chr20:–
1458206 GCCAGAGGT 1458200
1455719 AGGAGCAGGT 1455714
1455132 AGGTTTTATG 1455125
NM_001206736.1 (4) ^ 282

AA

amyloid beta (A4) precursor protein–binding, family B, member 3 (APBB3)

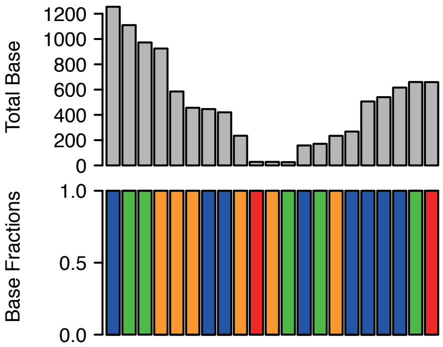

CAAGGGCCGTGACAGCCCCAT
UCSC XXX chr5:–
140562108 CAAGGGCCGGT 140562100
140561728 TACCCCAGCCCCAT 140561717
CCDS chr5:–
140562108 CAAGGGCCGGT 140562100
140561849 AGTGACAGGT 140561844
140561722 AGCCCCAT 140561717
NM_006051.3 (4) ^ 628

AB

amyloid beta (A4) precursor protein–binding, family B, member 1 (Fe65) (APBB1)

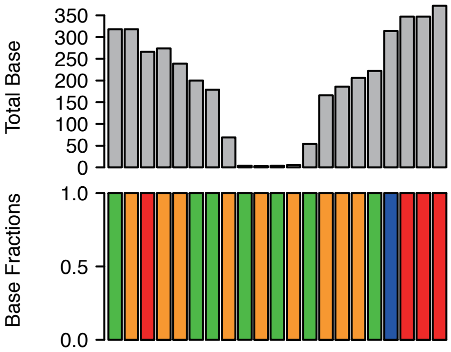

AGTGGAAGAGAGAGGGACTTT
UCSC XXX chr11:–
6402089 AGTGGAAGGTGGGA 6402078
6401690 ACAGGGACTTT 6401682
CCDS chr11:–
6402089 AGTGGAAGGT 6402082
6401982 AGAGAGAGGT 6401977
6401688 AGGGACTTT 6401682
NM_001257326.2 (5) ^ 608
NM_001257320.2 (6) ^ 608
NM_001257321.2 (4) ^ 608
NM_001164.4 (1) ^ 1385
NM_001257325.2 (7) ^ 680
NM_001257319.2 (9) ^ 725

AC

collagen, type XXV, alpha 1 (COL25A1)

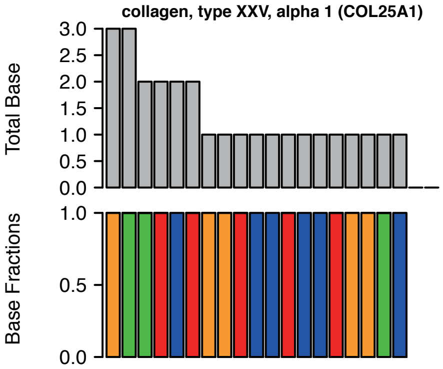

GAATCTGGTCCTCCTGGACAG
UCSC XXXX chr4:–
109050141 GAATCTGGTAAGTCTCT 109050127
109010375 AGGGACAG 109010370
CCDS chr4:–
109050141 GAATCTGGT 109050135
109048175 AGGTCCTCCTGT 109048168
109010375 AGGGACAG 109010370
NM_001256074.1 (3) ^ 416
NM_032518.2 (2) ^ 416
NM_198721.3 (1) ^ 416

AD

muscle, skeletal, receptor tyrosine kinase (MUSK)

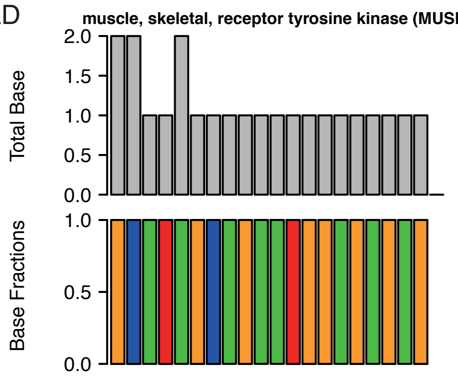

GCATAGCAGAATGGAGAGAGT
UCSC XXX chr9:+
110747792 GCATAGCAGGTAGGAT 110747805
110775786 TTAGAGAGT 110775792
CCDS chr9:+
110747792 GCATAGCAGGT 110747800
110762202 AGAATGGAGGT 110762208
110775788 AGAGAGT 110775792
NM_001166280.1 (2) ^ 945

Supplementary Figure S1 (continued).

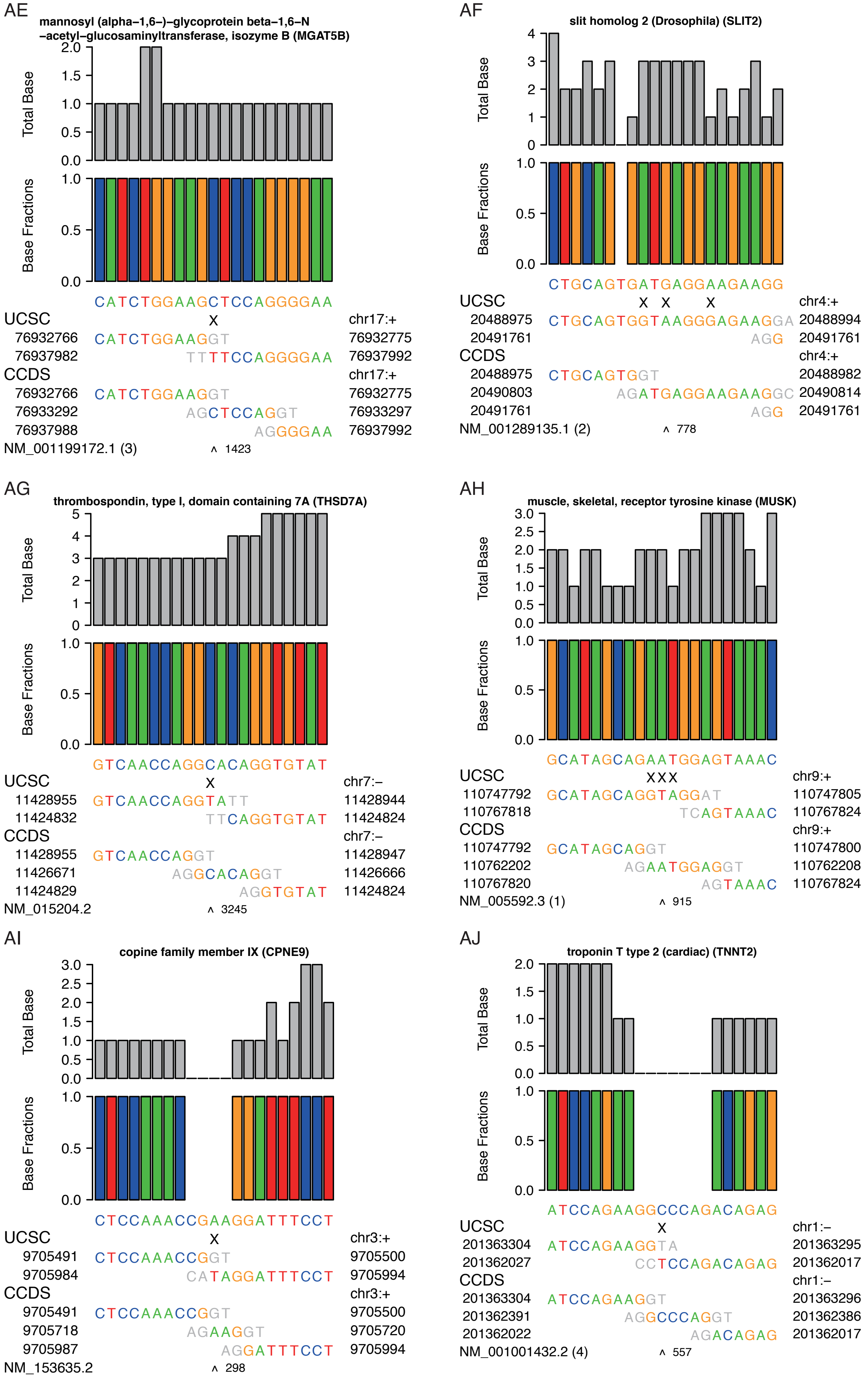

Supplementary Figure S1 (continued).

AK

solute carrier family 22 (organic anion transporter), member 7 (SLC22A7)

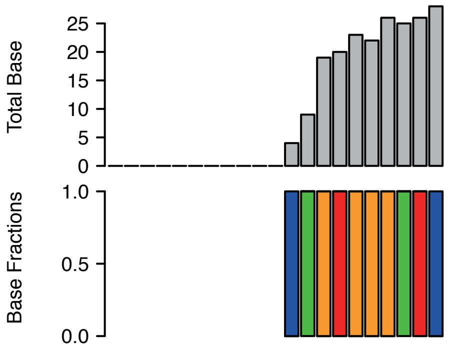

CAACTGAGTCCCAGTGGGATC
UCSC XX chr6:+
43298744 CAACTGAGGT 43298751
43299384 CCTTGCAGTGGGATC 43299396
CCDS chr6:+
43298744 CAACTGAGGT 43298751
43299092 AGTCCCAGGT 43299097
43299390 AGTGGGATC 43299396
NM_153320.2 (2) ^ 396

AL

synaptosomal–associated protein, 91kDa (SNAP91)

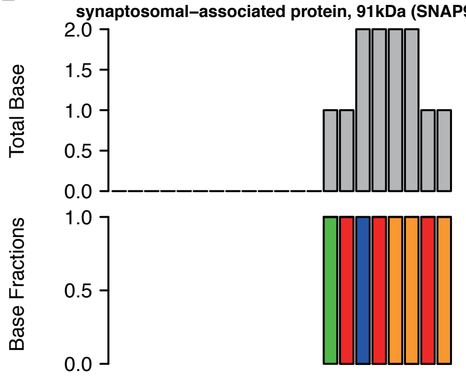

GAAACAATGAAGGATCTGGTG
UCSC XX X X chr6:–
83616975 GAAACAAGTACGCAT 83616963
83610677 AGATCTGGTG 83610670
CCDS chr6:–
83616975 GAAACAAGT 83616969
83614862 AGTGAAGGGT 83614857
83610677 AGATCTGGTG 83610670
NM_014841.2 (1) ^ 882
NM_001242792.1 (3) ^ 882

AM

echinoderm microtubule associated protein like 5 (EML5)

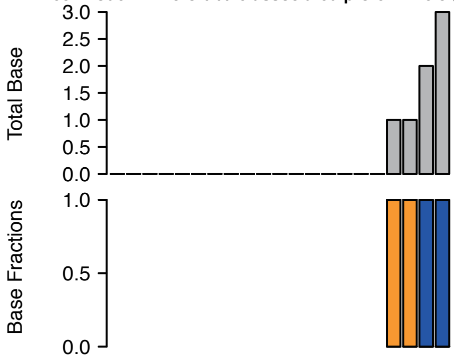

AGTAGTAAGAGGTTCCAGGCC
UCSC XX X chr14:–
88649920 AGTAGTAAGGT 88649912
88644520 CTCTGTTGCAGGCC 88644509
CCDS chr14:–
88649920 AGTAGTAAGGT 88649912
88646955 AGAGGTTCCAGGT 88646947
88644511 AGGCC 88644509
NM_183387.2 ^ 4021

AN

protocadherin–related 15 (PCDH15)

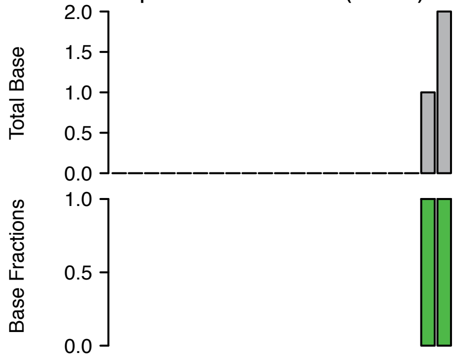

GCTCAATGCATAAGTATGAAA
UCSC X X X chr10:–
53827399 GCTCAATGTAAGT 53827389
53810666 ACAGGTATGAAA 53810657
CCDS chr10:–
53827399 GCTCAATGT 53827393
53825141 AGGCATAAGT 53825136
53810664 AGGTATGAAA 53810657
NM_001142771.1 (K) ^ 4386
NM_001142772.1 (L) ^ 4371

AO

unc–13 homolog C (C. elegans) (UNC13C)

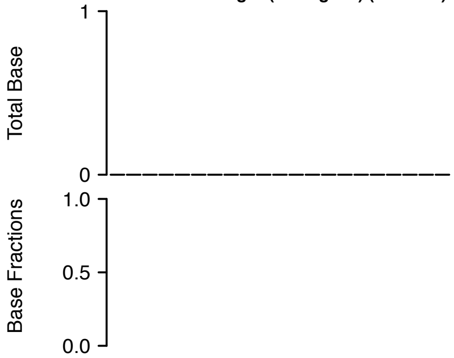

GAGATGTGGCCATGACCCTGG
UCSC XX X chr15:+
54235101 GAGATGTGGTAAGT 54235112
54237617 TTAGACCCTGG 54237625
CCDS chr15:+
54235101 GAGATGTGGT 54235108
54236430 AGGCCATGGT 54236435
54237619 AGACCCTGG 54237625
NM_001080534.1 ^ 3153

AP

neural cell adhesion molecule 1 (NCAM1)

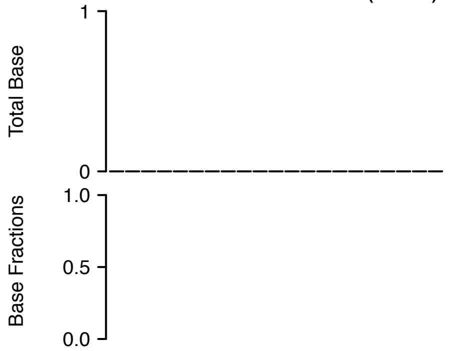

CAGCCAGTCCAAGGGGAACCC
UCSC X chr11:+
113235155 CAGCCAGTCCGT 113235164
113255874 TTCAGGGGAACCC 113255884
CCDS chr11:+
113235155 CAGCCAGTCCGT 113235164
113246368 AGAAGGT 113246370
113255877 AGGGGAACCC 113255884
NM_181351.4 (2) ^ 1826
NM_001242608.1 (4) ^ 1796
NM_001242607.1 (5) ^ 1904
NM_000615.6 (1) ^ 1796

Supplementary Figure S1 (continued).

AS

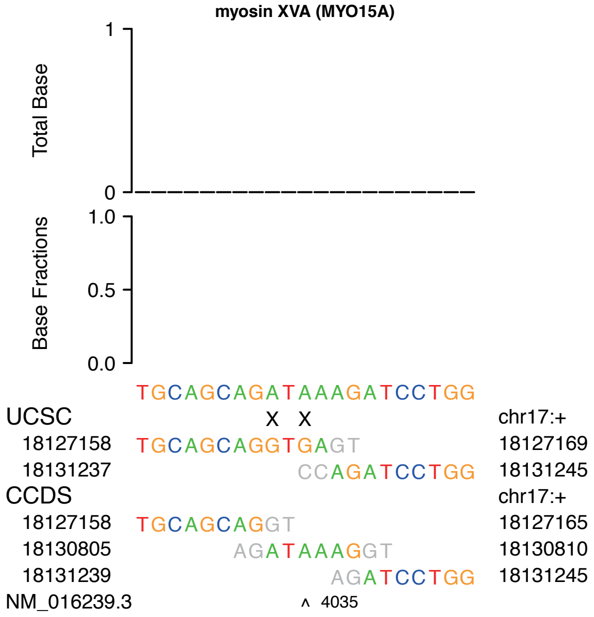

Supplementary Fig. S1. Differences in exon definitions by UCSC and CCDS annotations for those mRNAs that are classified as class 1 (exactly match) by CCDS but class 2 (substitution) by UCSC. Read counts of pooled GEUVADIS RNA-seq (top), base frequencies (middle) and mRNA sequences and exon sequences defined by UCSC and CCDS (bottom) are shown. Mismatches between mRNA and coding sequences are marked by “X”. The gene names are shown as the title of each panel.

Supplementary Figure S2.

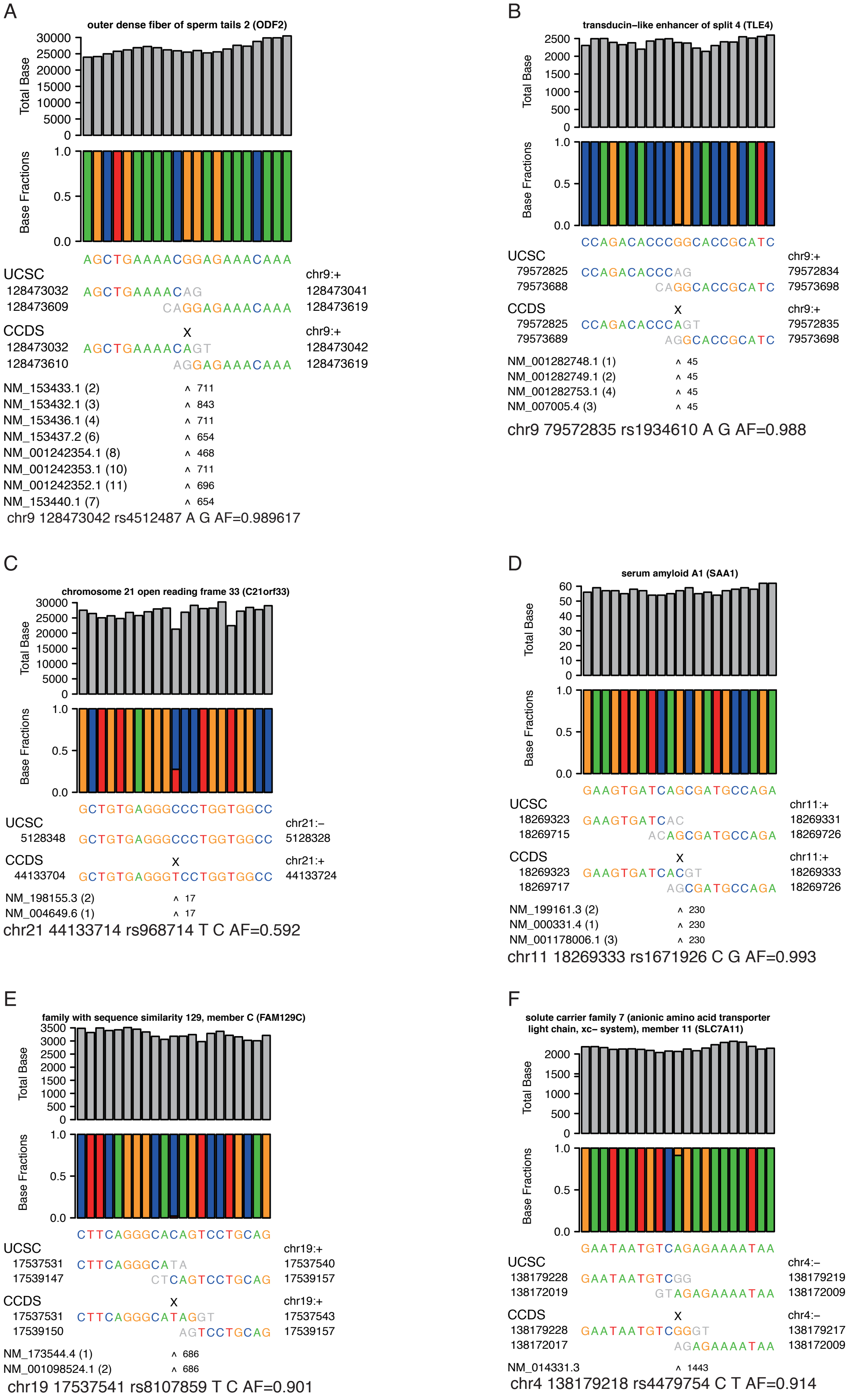

Supplementary Fig. S2. Differences in exon definitions by UCSC and CCDS annotations for those mRNAs that are classified as class 1 (exactly match) by UCSC but class 2 (substitution) by CCDS. Read counts of pooled GEUVADIS RNA-seq (top), base frequencies (middle) and mRNA sequences and exon sequences defined by UCSC and CCDS (bottom) are shown. Mismatches between mRNA and coding sequences are marked by “X”. The gene names are shown as the title of each panel. The SNPs explaining the base mismatch by the CCDS alignment are shown in the lowest row in each panel.

Supplementary Figure S3

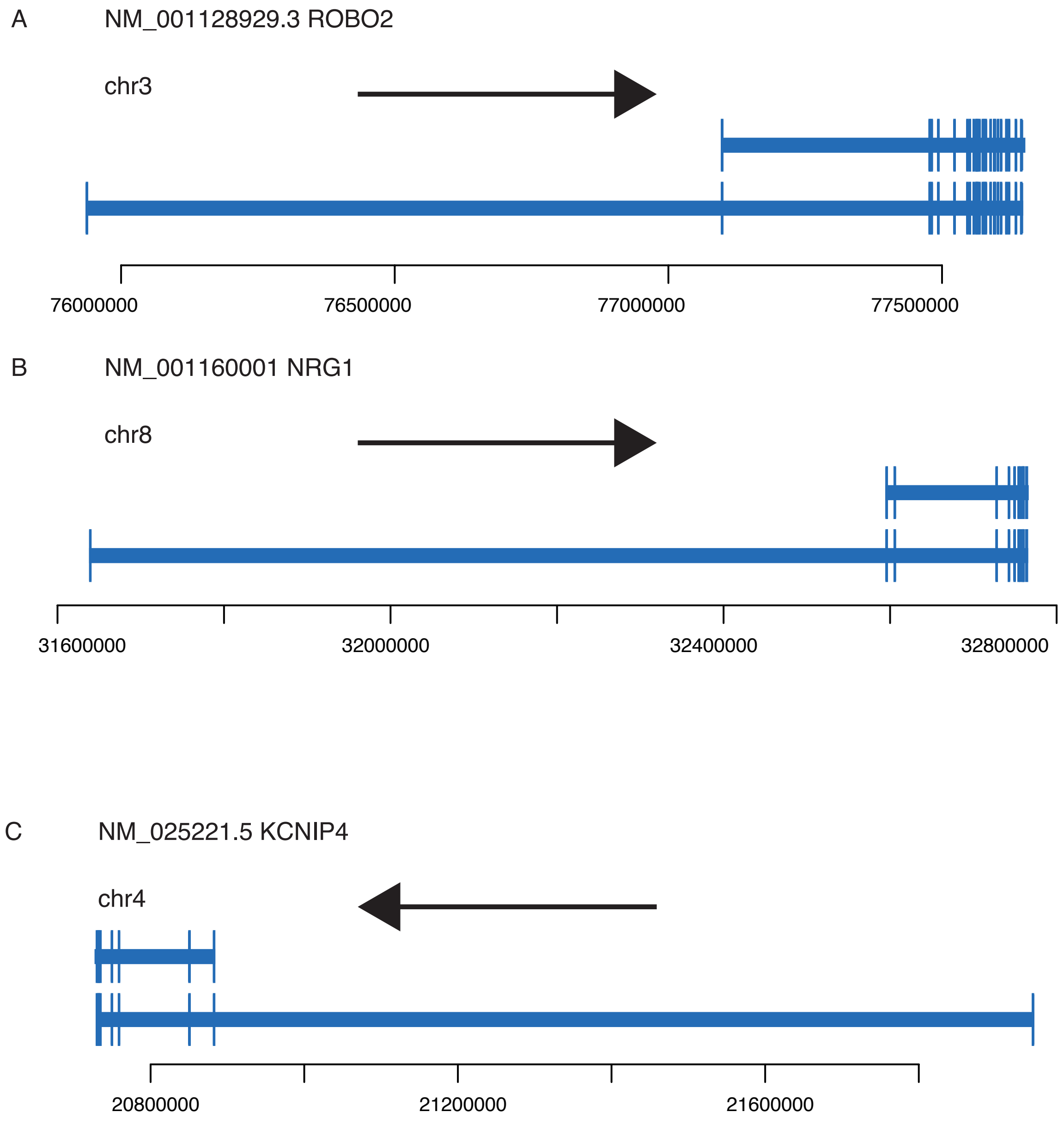

Supplementary Fig. S3. The exon-intron definitions by UCSC (top) and CCDS (bottom) for the A) ROBO2, B) NRG1 and C) KCNIP4 genes.

Supplementary Data 1

A list of the discordant SNVs with functional annotations by GWAS catalogue, ClinVar and UniProt FN record.

Supplementary Data 2

The full list of concordance and discordance for the SNVs in 1K genomes data.
